# High-throughput LDI MS technology decodes the distinct metabolic landscape of prostate cancer in a large-scale cohort

**DOI:** 10.1186/s40364-025-00804-z

**Published:** 2025-07-09

**Authors:** Xinrong Jiang, Chen Zhang, Jing Le, Jie Zhang, Shuo Cao, Xinran Xu, Xiaoming Chen, Sheng Cheng, Haitao Yu, Haofei Jiang, Ruichen Zang, Kunyu Wang, Weiwu Chen, Haobo Fan, Jianmin Wu, Yanlan Yu, Guoqing Ding

**Affiliations:** 1 Department of Urology, School of Medicine, Sir Run Run Shaw Hospital, Zhejiang University, Hangzhou, 310016 P. R. China; 2 Institute of Analytical Chemistry, Department of Chemistry, Zhejiang University, Hangzhou, 310058 P. R. China; 3 Well-healthcare Technologies Co., Hangzhou, 310051 P. R. China; 4 Clinical Laboratory, Sir Run Run Shaw Hospital, School of Medicine, Zhejiang University, Hangzhou, 310016 P. R. China

**Keywords:** Metabolomics, Prostate cancer, Benign prostatic hyperplasia, Non-invasive diagnosis, Laser desorption/Ionization mass spectrometry

## Abstract

**Background:**

Prostate cancer (PCa) remains a leading global malignancy, yet current diagnostic reliance on prostate-specific antigen (PSA) testing is limited by suboptimal sensitivity and specificity for early-stage detection. The present study aims to establish an effective high-throughput screening technique for accurate PCa diagnosis.

**Methods:**

A large-scale cohort of 28,892 subjects was considered for inclusion in this study, and 1133 volunteers were finally selected, including 600 healthy controls, 160 patients diagnosed with other diseases of urinary system, 89 patients diagnosed with benign prostate hyperplasia (BPH), and 284 PCa patients. Discovery and internal validation phases of diagnostic models were conducted through machine learning of urine metabolic fingerprints obtained by laser desorption/ionization mass spectrometry (LDI-MS). Furthermore, the developed diagnostic model was verified in an external validation cohort.

**Results:**

In retrospective cohort, the stepwise binary classification model achieved satisfactory diagnostic performance with areas under curves (AUCs) of 0.9599–0.9957 in the discovery (*n* = 567) and internal validation dataset (*n* = 284). In the external validation cohort (*n* = 282), AUC values from the ROC curves that differentiate Non-PD from PD, BPH from PCa, and HC from UD were 0.9815, 0.9705, and 0.9980, respectively. More than 95% significant PCa patients in the gray area (3 < tPSA < 10 ng/mL) were successfully identified from BPH subjects. Notably, four metabolite-related candidate genes were identified in this work, including *AOX1*, *PON3*, *CBS* and *ASPA*.

**Conclusions:**

This study demonstrated the clinical potential of an LDI-MS-based non-invasive urine biopsy for early prostate cancer detection, particularly in improving diagnostic accuracy for patients with tPSA levels in the gray zone (3–10 ng/mL).

**Supplementary Information:**

The online version contains supplementary material available at 10.1186/s40364-025-00804-z.

## Introduction

Prostate cancer accounts for the fifth leading cause of cancer-related mortality in men worldwide [1]. Considering that PCa has a relatively long latency period, it is critical to establish an efficient screening method for its early detection [2]. In the early stage, PCa has no special clinical manifestations. Currently, PCa diagnosis mainly relies on the quantification of PSA [3]. Unfortunately, this method may lead to overdiagnosis and overtreatment because it is prostate-specific rather than cancer-specific [4, 5]. PSA levels were also influenced by other factors, such as urinary retention and urinary tract infection, resulting in inevitable false-positive results [6]. Histopathological evaluation is mandatory for a final diagnosis [2]. However, prostate biopsy is an invasive procedure that might cause serious complications for patients [7]. Current method limitations drive intense focus on molecular biomarkers for more accurate PCa diagnosis.

Urine, as a recycler of endogenous metabolites, is an appealing source for mining non-invasive biomarkers [8]. Diseases of urinary system directly affect the composition of urine samples, PCa-related substances (such as DNA, RNA, metabolites) may be excreted into the urine [9]. In recent years, some potential biomarkers, such as cfDNA, have been mined in biofluids to assist PCa screening [10–13]. cfDNA analysis requires sophisticated techniques due to low levels and damage susceptibility [14]. The optimal combination of miRNAs for PCa diagnosis needs to be further confirmed [15]. Recent studies have provided unbiased evidence to elucidate the strong correlation between metabolite fluctuation and PCa phenotype [16, 17]. Unfortunately, the presence of interfering contents such as numerous background salts in urine may hamper the metabolite extraction for mass spectrometry-based analysis [18]. To facilitate the effective extraction of urinary metabolites, our laboratory has proposed a tip-contact extraction (TCE) method coupled with laser desorption/ionization mass spectrometry (LDI-MS) platform, which has been confirmed applicable for ultrasensitive and reproducible metabolite detection of biofluids [19, 20].

In this study, we aimed to identify metabolic signatures associated with PCa and construct an effective high-throughput PCa screening technique based on the LDI-MS platform. Nontargeted metabolomics was performed on urine samples from healthy controls (HC) and patients diagnosed with PCa, BPH or other diseases of urinary system (UD). Multivariate statistical analysis screened dysregulated metabolites as unique biomarkers and attractive targets to speculate potential molecular mechanisms. With the assistance of machine learning, a stepwise prediction model was well-trained, which showed high accuracy in distinguishing PCa patients from other subjects.

## Methods

### Participant recruitment

Participants were recruited consecutively in the department of urology from Sir Run Run Shaw Hospital of Zhejiang University between 2021 and 2023. The following groups were defined according to the EAU-EANM-ESTRO-ESUR-SIOG Guidelines on Prostate Cancer [21]: HC group included male volunteers with negative urine routine results and no history of any other metabolic disease; UD group included male patients diagnosed with other urinary diseases, such as urinary calculi, glandular cystitis, renal cell carcinoma and bladder cancer; BPH group included patients with benign prostatic hyperplasia and no history of any other malignancies; PCa group included patients with prostate cancer confirmed by histopathology. Among them, BPH and PCa were classified as prostate diseases (PD) and confirmed by pathological examination. All non-PD (HC and UD) participants were confirmed by medical examination results and consulting medical history. Notably, all patients did not receive any anti-tumor treatment prior to urine sample collection. To reduce the interference of confounding factors such as age, body mass index (BMI), drinking habits, smoking status, chronic diseases and so on, propensity score matching was performed. In the PCa group, clinically significant cancer (Gleason score ≥ 7) accounted for more than 80%, and PSA threshold was defined by the National Comprehensive Cancer Network guidelines [22]. The clinical characteristics of all participants are shown in Table S1-S3.

### Urine collection and Preparation

According to the urine sampling guidelines [23], the midstream of first-morning urine was uniformly collected before the initial biopsy with an empty stomach at around 7:00 to 9:00 a.m. After collection, urine samples were immediately centrifuged to remove the cell debris and insoluble residues (8000 g for 10 min at 4 °C) and stored at − 80 °C until use. Prior to MS analysis, urine samples were thawed on ice and fluorinated ethylene propylene coated silicon nanowires (FEP@SiNWs) chips were prepared via the one-step metal assisted chemical etching method [24]. Urinary metabolites were effectively extracted onto FEP@SiNWs chips through TCE method, which has been described in our previous work [20]. The Ethical Committee of the Sir Run Run Shaw Hospital of Zhejiang University approved the protocol (No. 20230257) and the study was performed in accordance with the ethical standards laid down in the 1964 Declaration of Helsinki and its later amendments. Written informed consent was obtained from each patient.

### Statistical analysis

Data acquisition and processing of the raw mass spectra were performed by FlexAnalysis (Bruker Daltonics Co.) and ClinProTools (Bruker Daltonics Co.) was utilized to output the list of peaks with S/*N* > 3. Data normalization was conducted by R 3.5.2 software with the cubic spline method. Student’s t-test was performed using the ttest2 function in MATLAB for pairwise analysis. In order to ensure the statistical detection ability, false discovery rate (FDR) was calculated via the p.adjust function based on the Benjamini-Hochberg method. Differential peaks along with group information were imported into SIMCA software for supervised orthogonal partial least squares-discriminant analysis (OPLS-DA) to reveal intergroup differences. Higher values of R²X (explained variance in X), R²Y (explained variance in Y), and Q² (predictive ability) indicate greater reliability of the constructed model.

## Results

### Study design and quality control assessment

**Fig. 1:**
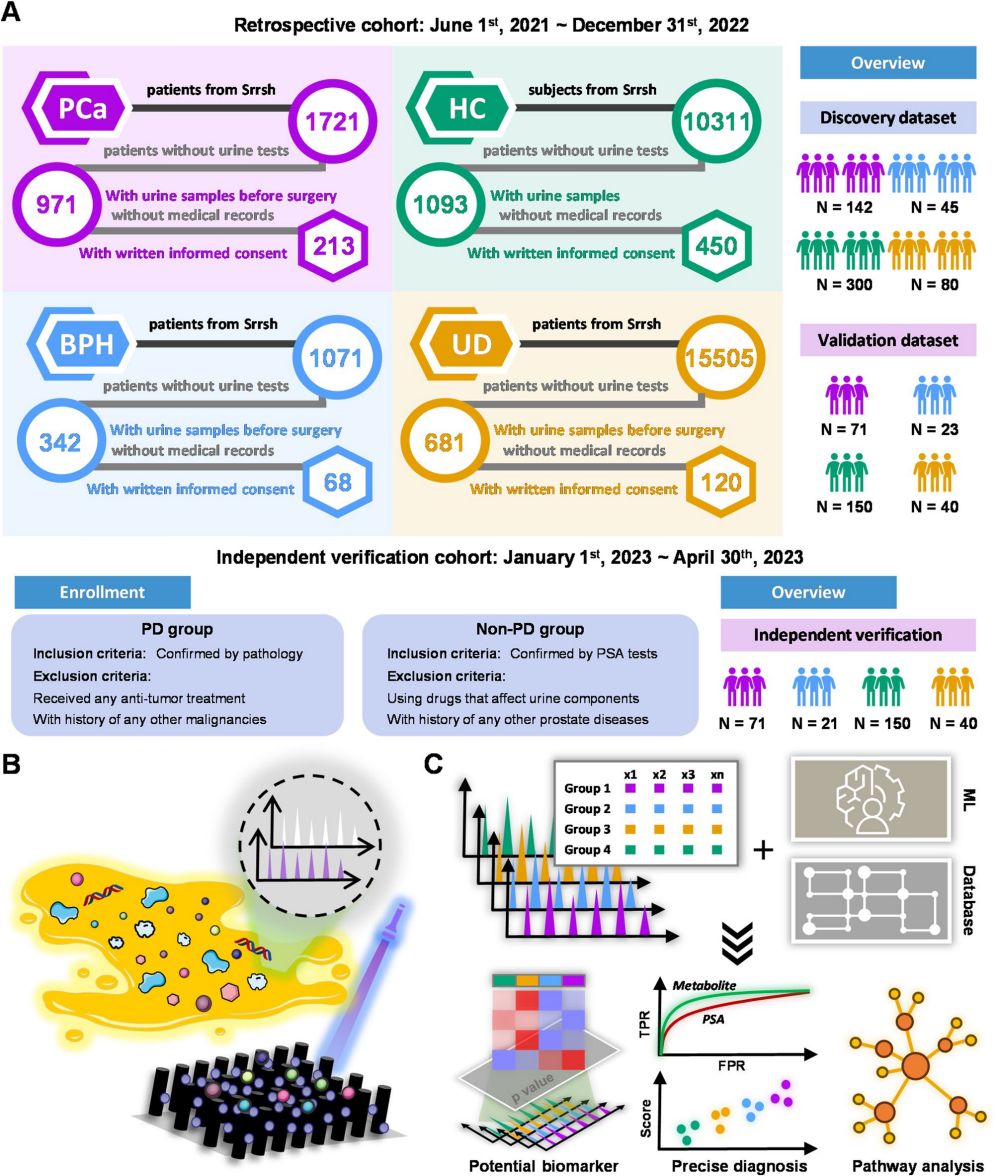
Schematics of urine metabolomics for prostate cancer diagnosis and accurate disease discrimination. (**A**) The design of cohort recruitment. The retrospective cohort was consisted of 851 subjects enrolled from June 1st, 2021 to December 31st, 2022, while the independent verification cohort included 282 subjects from January 1st, 2023 to April 30th, 2023. (**B**) Non-targeted metabolomic profiling on LDI-MS platform. (**C**) Potential biomarker discovery, precise PCa diagnosis, and disturbed pathway analysis were achieved with the assistance of machine learning and open-source database

As shown in Fig. 1A, a large-scale cohort of 18,297 patients and 10,311 healthy volunteers who visited the Department of Urology at Sir Run Run Shaw Hospital from June 1st, 2021 to December 31st, 2022 was initially considered for inclusion in the discovery and internal validation phases of diagnostic model. However, 9218 healthy controls, 750 PCa, 729 BPH, and 14,824 UD patients were excluded due to the lack of preoperative urine samples and urine routine results. Ultimately, 213 PCa, 68 BPH, and 120 UD patients agreed to participate in this study. Based on propensity score matching (PSM), 450 healthy volunteers who signed informed consent forms were selected as the control group. According to the well-defined criteria, 282 participants from January 1st, 2023 to April 30th, 2023 were independently selected in the external validation cohort. Propensity scores in the disease and control groups showed a more similar distribution after matching (Figure S1). Specifically, the discovery and internal validation sets were retrospectively and randomly created in a 2:1 proportion. The procedure for metabolomic profiling on the LDI-MS platform is presented in Fig. 1B. Through the good charge transfer ability of SiNWs and the phase transition of initiators, urinary metabolites adsorbed onto FEP@SiNWs chip were sensitively detected. To ensure the reliability of acquired mass spectra, a pooled quality control (QC) sample was prepared and measured in each independent batch of experiments. As shown in Figure S2, the median RSD of intra-batch and inter-batch QC measurements was 7.6% and 14.6%, respectively. In addition, the metabolic profiles of QC samples collected in each independent batch of experiments were clustered together (Figure S3A). Subsequently, we calculated the errors of the X and Y axes in the OPLS-DA plot for QC samples. As shown in Figure S3B, C, the errors of QC samples all fell within the 3σ range, demonstrating acceptable stability. Moreover, multivariate statistical analysis was conducted for potential biomarker discovery, precise PCa diagnosis, and disturbed pathway analysis. Notably, we built prediction models based on differential metabolites with the assistance of machine learning, which may enable mass population screening (Fig. 1C).

### Metabolomic landscape of prostate cancer and controls

**Fig. 2:**
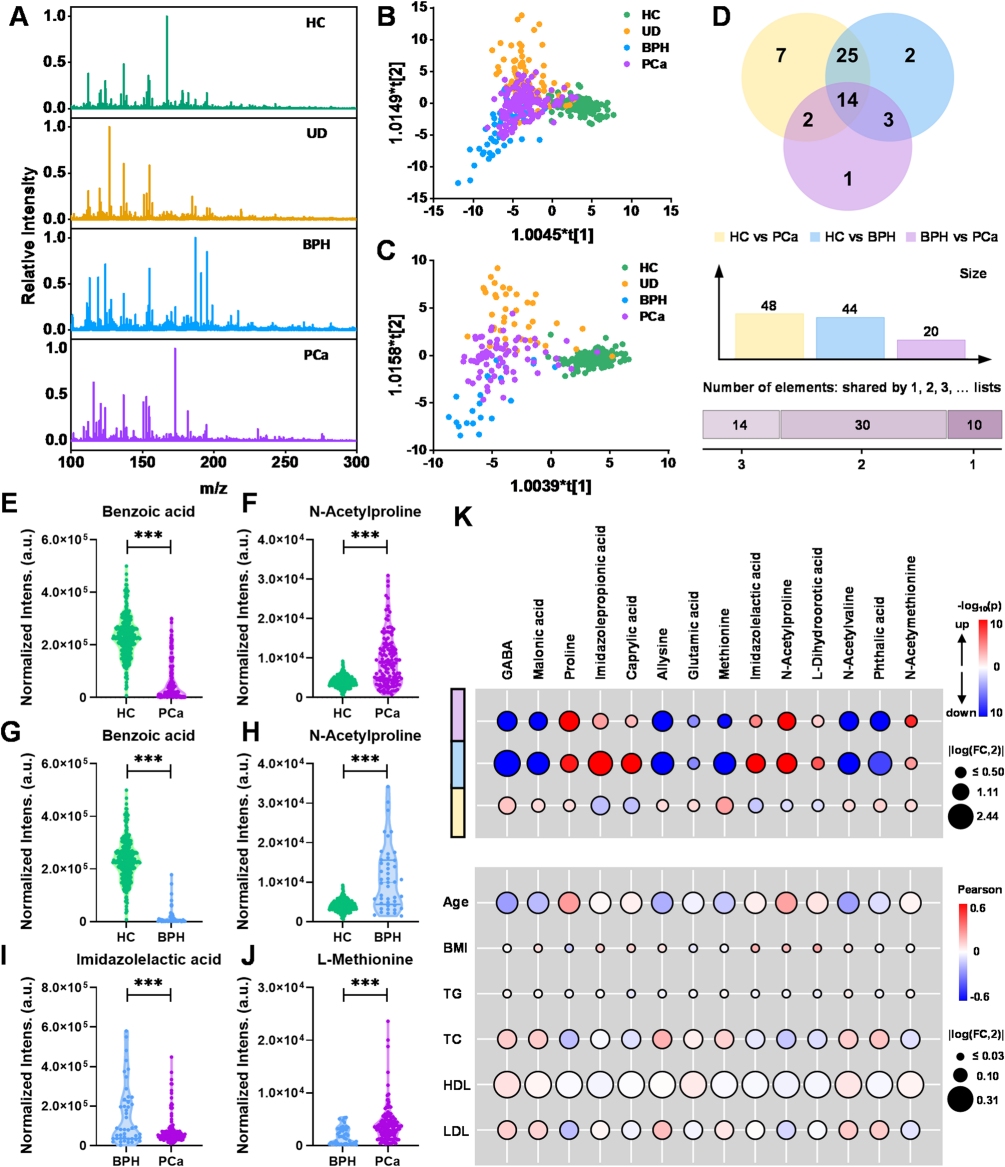
Metabolomics landscape of urine samples from BPH, PCa patients and healthy controls. (**A**) Representative LDI mass spectra of urine samples collected from different groups. (**B**, **C**) The OPLS-DA result for discrimination between BPH, PCa, UD and healthy controls in the discovery (R²X = 0.42, R²Y = 0.64, Q² = 0.67) and internal validation (R²X = 0.45, R²Y = 0.75, Q² = 0.78) cohorts, respectively. (**D**) Venn diagrams represent dysregulated metabolites in pairwise analysis, respectively. (**E-J**) Representative box plots of top up- or down-regulated metabolites in pairwise analysis, respectively. (**K**) Visualization of changes in certain molecules or clinical factors between two groups. In the left ribbon, purple, blue and yellow represent pairwise analysis between BPH and PCa, HC and BPH, HC and PCa, respectively

Through untargeted metabolomics analysis, around 297 peaks could be reliably detected across all urine samples. Figure 2A displayed the normalized MS spectra of HC, UD, BPH and PCa patients, and the different metabolome landscapes in the low molecule-weight region provided the possibility for subsequent discrimination. As shown in OPLS-DA plots, a certain separation was achieved between four groups (Fig. 2B, C). In order to obtain the differential metabolites that are intimately associated to each phenotype, pairwise analysis between groups was performed, including HC vs. PCa, HC vs. BPH, and BPH vs. PCa. The detailed information of dysregulated signatures was summarized in Table S5-S7, and the overlap of identified metabolites in each pairwise analysis was shown in Fig. 2D. Especially, fourteen metabolites were uncovered to be differentially expressed across all PD groups (adjusted p value < 0.05). As shown in Fig. 2E-J, violin plots exhibited the most up- and down-regulated metabolites in each pairwise analysis, including benzoic acid, N-Acetylproline, imidazolelactic acid, and methionine. The detailed identification information of altered metabolites was summarized in Table S8-S9 and Figure S4. Figure 2K visualized the log2-transformed fold change and corresponding p-value of certain metabolites and clinical factors in pairwise analysis. As shown in the bubble plot, those differentially expressed signatures had relatively weak correlations (| r| < 0.3) with clinical parameters, which ensured the diagnostic efficiency of identified metabolites.

### Dysregulated metabolic pathways in prostate cancer and benign prostatic hyperplasia

**Fig. 3:**
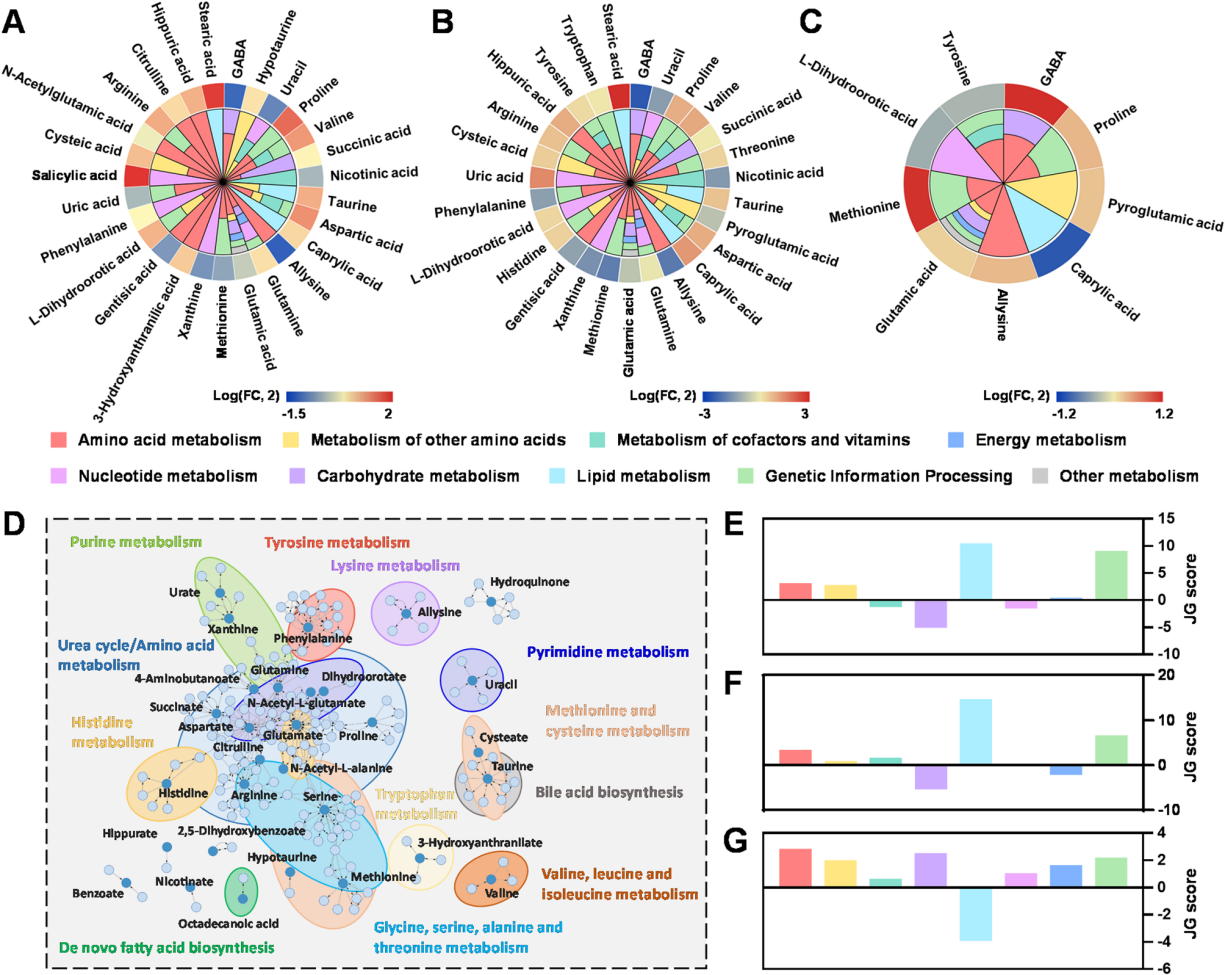
Dysregulated biological pathways hidden in different phenotypes. (**A-C**) The role of altered metabolites in corresponding pathway categories. The pie size is proportional to the number of involved pathways. (**A**) HC vs. PCa (**B**) HC vs. BPH (**C**) BPH vs. PCa. (**D**) Network analysis of PCa-related metabolites shows the disturbance in metabolic pathways. The dark blue and light blue nodes represent differential metabolites and their closely related metabolites, respectively. (**E-G**) Relative enrichment of the major metabolic pathways in pairwise analysis between HC and PCa (**E**), HC and BPH (**F**), BPH and PCa (**G**), represented by JG score

With the assistance of open-source databases, the disturbed metabolic pathways in each phenotype were further investigated. As shown in Fig. 3A-C, the role and significance of altered metabolites in the corresponding pathway categories were mapped through KEGG database. In each pie chart, amino acid metabolism occupied a considerable space, which demonstrated that most of differential signatures might derived from metabolic disorders of amino acids. Besides, network analysis was performed to picture the metabolite-metabolite associations among PCa-associated metabolites. As shown in Fig. 3D, metabolic disturbances were mainly concentrated in purine metabolism, pyrimidine metabolism, urea cycle and amino acid metabolism. Furthermore, JG score [25] was calculated to statistically measure the perturbation of certain metabolic pathway categories in each phenotype (Fig. 3E-G). As shown in Fig. 3E, F, PCa and BPH exhibited the same trend in most categories compared to healthy controls. When compared to BPH, most metabolic processes were upregulated in PCa patients (Fig. 3G). Finally, eighteen metabolic pathways were found to be disturbed across three groups by intersection pathway analysis (Figure S5). Figure S6 showed the JG score and corresponding p-value of those pathways, in which map00970 (Aminoacyl-tRNA biosynthesis) and map00220 (Arginine biosynthesis) ranking at the top.

**Fig. 4:**
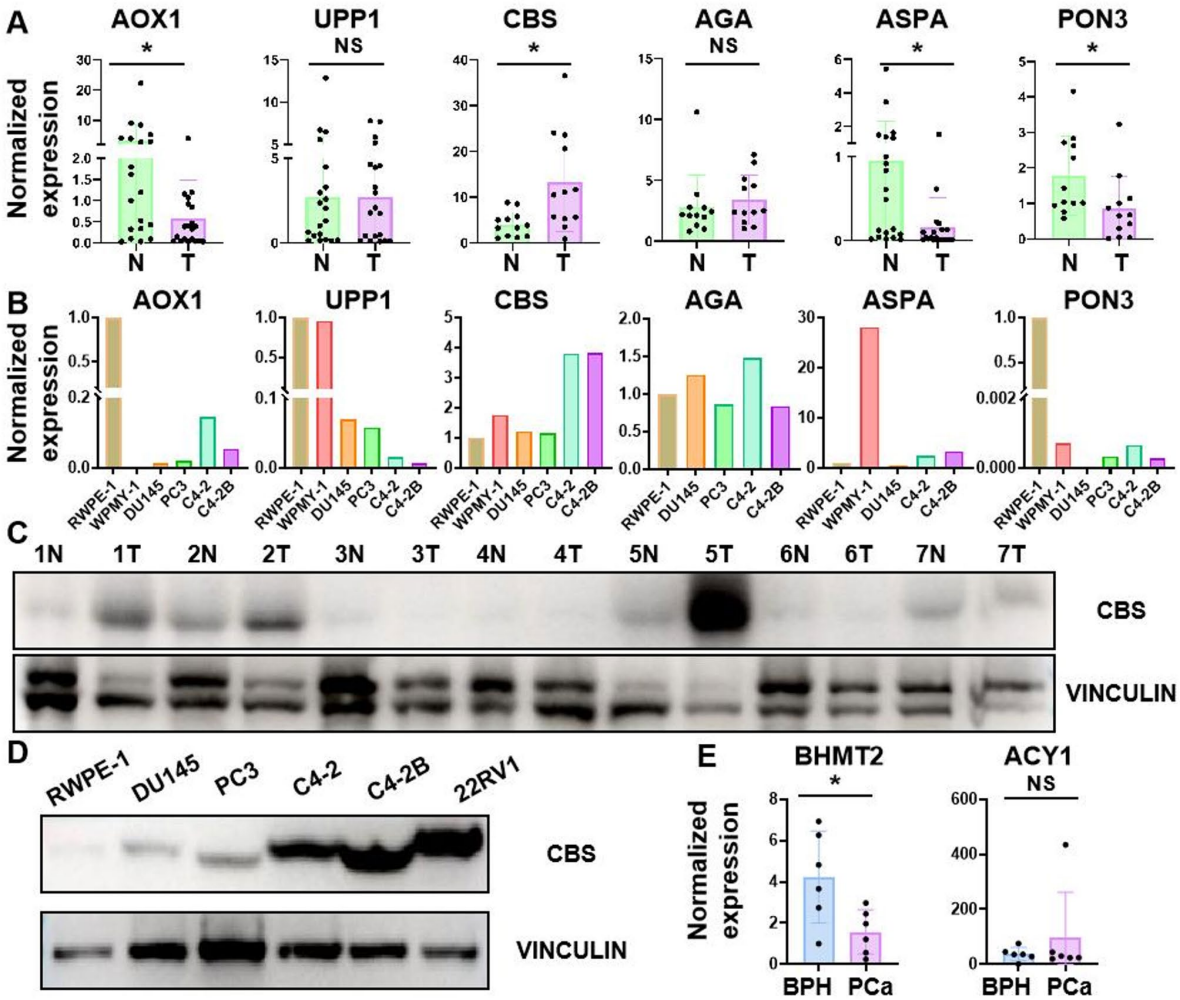
Biological experiments for the validation of differential metabolite-related candidate genes. (**A**) mRNA levels of metabolite-related genes in tumor and adjacent normal tissue samples from PCa patients. *n* = 20 (**B**) mRNA levels of metabolite-related genes in normal prostate epithelial cell line and prostate cancer cell lines such as PC3, DU145, C4-2 and C4-2B. (**C**, **D**) the protein level of *CBS* in prostate cancer patients’ tissues (**C**) and prostate cancer cell lines (**D**) by Western blot. (**E**) mRNA levels of metabolite-related genes in tissue samples from patients with benign prostatic hyperplasia

To further validate the metabolic disturbances observed in urine samples, transcriptome analysis was achieved based on RNA-Seq data from the open-source TCGA database. As shown in Figure S7, there were 681 upregulated genes and 2326 downregulated genes in PCa. In addition, KEGG functional enrichment analysis demonstrated that the altered metabolic pathways were concentrated into nucleotide metabolism and amino acid metabolism (Figure S8A). Impressively, the metabolic disorders discovered in tissue transcriptomics have been mirrored in urine metabolomics in this work (Figure S8B). Network analysis of PCa-associated metabolites indicated that 176 genes were involved in the metabolism of differential features (Figure S9). Through mRNA expression analysis of GEPIA website [26]twenty-three metabolite-related genes were found to be abnormally expressed in PCa, including *AOX1*, *UPP1*, *PON3*, *CBS*, *AGA*, *ASPA* and so on (Figure S10). Subsequently, Kaplan-Meier curve and clinical significance of candidate genes were depicted from the UALCAN website [27] (Figure S11-S13). Besides, we have reviewed and summarized several Asian databases to confirm the perturbation of significant genes (Figure S14). To determine the biological relevance of these genes above in PCa, we collected tumor and adjacent normal tissue samples from PCa patients who underwent radical prostatectomy. Interestingly, we found that *AOX1*, *CBS*, *ASPA* and *PON3* were significantly consistent with our analysis (*n* = 20, Fig. 4A). However, *UPP1* and *AGA* had no obvious biological correlation. Meanwhile, we explored the mRNA levels of these genes in normal prostate epithelial cell lines and PCa cell lines such as PC3, DU145, C4-2 and C4-2B (Fig. 4B). Furthermore, we searched the protein levels of *CBS* in PCa cell lines and tissues by Western blot, and significantly increased expression of *CBS* in prostate cancer was observed (Fig. 4C, D).

**Fig. 5:**
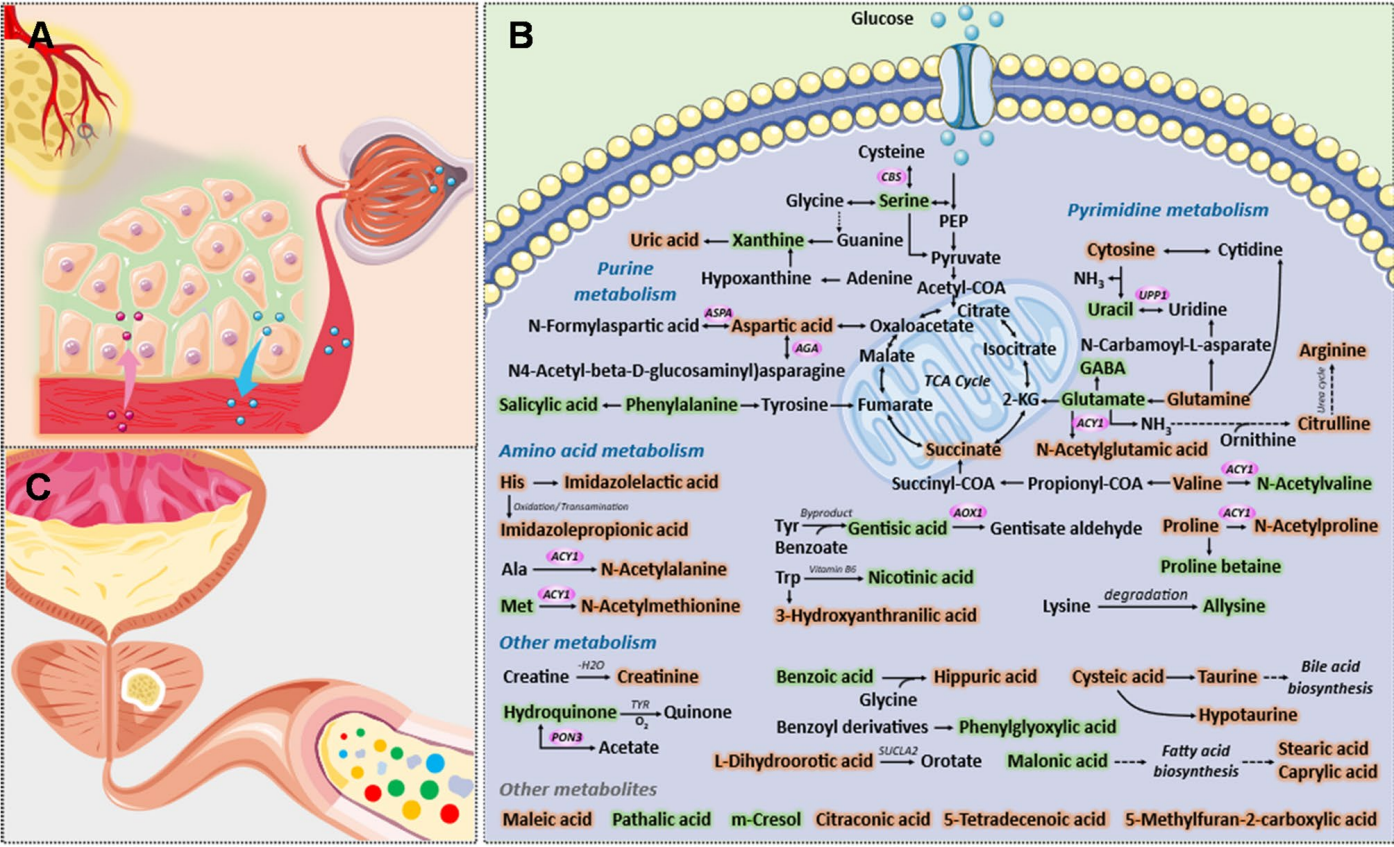
(**A**) Metabolites secreted by tumor cells are filtered through the glomeruli into urine. (**B**) The prostate affects urine excretion. (**C**) Pathway map of PCa-related metabolites and genes. Green and orange indicate metabolites that are down-regulated or up-regulated in PCa patients compared to healthy controls, and differentially expressed genes are marked with red circles

Similarly, a metabolite-reaction-enzyme-gene network was constructed based on the differentially expressed metabolites between PCa and BPH groups (Figure S15A). Among all metabolite-related genes, three significant genes were dysregulated in the GSE30994 dataset, including *NAGS*, *BHMT2* and *ACY1* (Figure S15B-D). mRNA analysis of real tissue samples showed that *BHMT2* was consistent with our results, while *ACY1* did not have a good biological correlation (Fig. 4E). Finally, a pathway map was pictured to display the perturbations of metabolites and related genes in PCa patients. On the one hand, metabolites secreted by tumor cells are filtered through the glomeruli into the urine; on the other hand, the prostate, as an organ of the urinary system, affects urine excretion [28] (Fig. 5A, B). Therefore, we have good reason to believe that metabolic fluctuations observed in urine may mirror the metabolic dysregulation of PCa cells (Fig. 5C).

### Evaluation of different machine learning algorithms

**Fig. 6:**
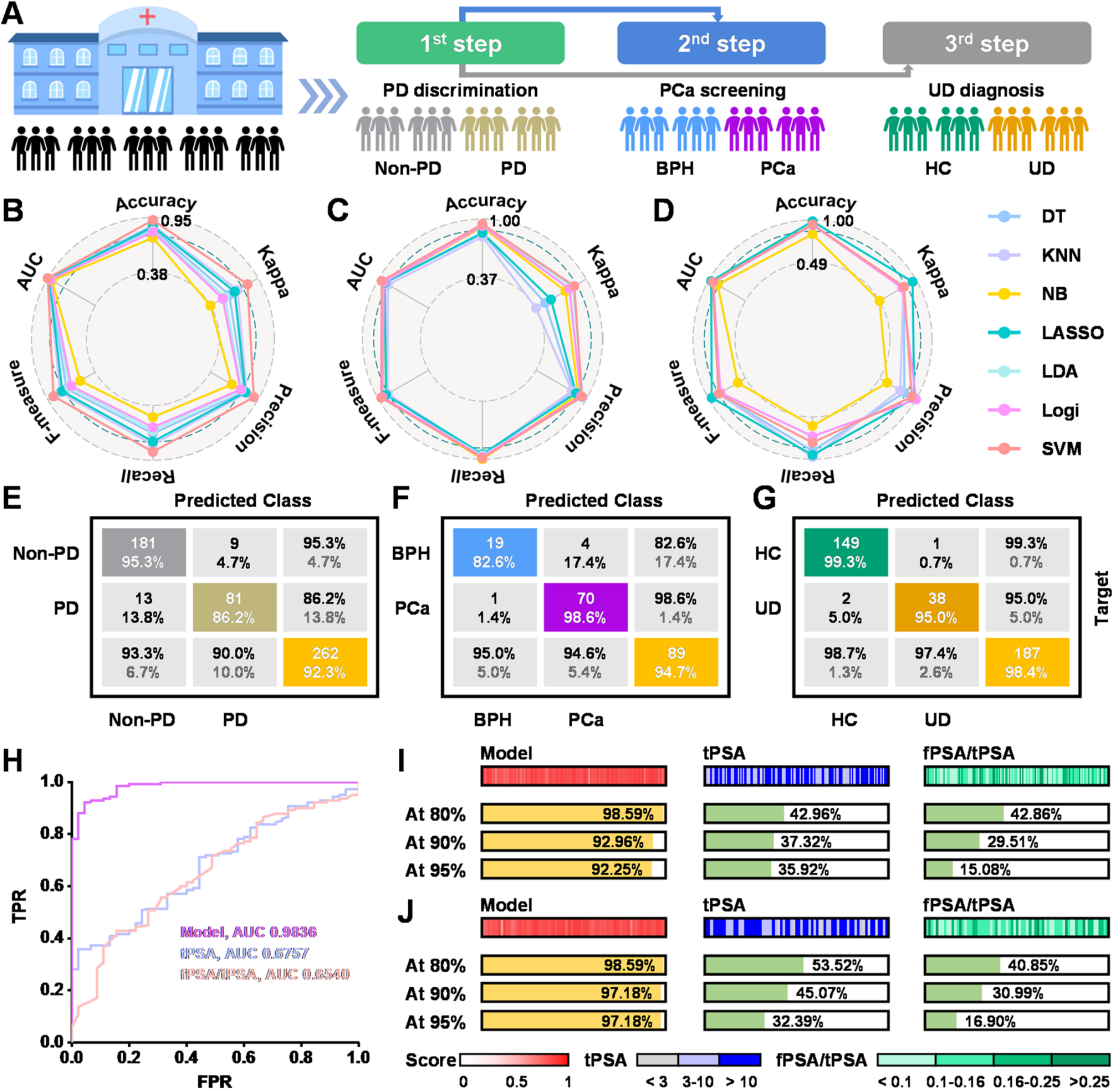
Accurate PCa diagnosis based on urine metabolomics with the assistance of machine learning. (**A**) Concept of three-step predictive model. (**B-D**) Radar map displays the classification performance of different machine learning algorithms for each step prediction in the internal validation dataset, evaluated by Kappa statistic, accuracy, F-measure, AUC, and precision. (**B**) Non-PD vs. PD (**C**) BPH vs. PCa (**D**) HC vs. UD. (**E-G**) Confusion matrix for the first (**E**), second (**F**) and third (**G**) step predictions based on the optimized model. (**H**) Receiver operating curves for PCa diagnosis in the discovery set. (**I**, **J**) Detection rates of PCa group at different specificities in the discovery dataset (**I**) and internal validation dataset (**J**)

To address the class imbalance among the four categories in this study and prevent minority class from being overwhelmed by majority classes, we employed stepwise binary classification rather than multiclass classification for our machine learning modeling. Stepwise binary classification decomposes the multiclass problem into multiple binary classification subtasks, thereby simplifying the learning of decision boundaries. Besides, stepwise binary classification can more flexibly address class imbalance issues, allowing for the selection of different feature subsets for each binary classification task and thereby improving the distinguishability between individual class pairs. Figure 6A showed the schematic illustrations of the three-step diagnostic model, including PD discrimination, PCa screening and UD diagnosis.

Notably, seven classification and regression algorithms have been discussed in this work, including decision trees (DT), nearest neighbor classifier (KNN), naïve Bayesian classifier (NB), LASSO regression, linear discriminant analysis (LDA), logistic regression (Logi), and support vector machine (SVM). DT recursively partitions the feature space via a tree-like structure, capable of handling nonlinear relationships, making it suitable for datasets with moderate sample sizes and feature dimensions. KNN performs classification by measuring sample proximity, making it particularly suitable for local pattern recognition in low-dimensional datasets with balanced class distributions. NB operates under the feature independence assumption and computes probabilities via Bayes’ theorem, demonstrating robust performance in small-sample and high-dimensional scenarios. LASSO regression applies a penalty function to compress high-dimensional variables, retaining only the most significant features for model fitting while maintaining strong interpretability. LDA and Logi serve as benchmark methods for binary classification, particularly suited for low-to-moderate dimensional metabolite data after feature selection. SVM employs kernel functions and maximum-margin hyperplanes to solve complex classification problems. It performs exceptionally well with small-sample, high-dimensional data, but is sensitive to class imbalance and parameter tuning. Kappa statistic, accuracy, F-measure, precision, recall and the area under the curve (AUC) were adopted to measure the diagnostic efficiency of different models for each step prediction.

As illustrated in Fig. 6B-D, those models constructed by SVM, SVM and LASSO regression demonstrated optimal classification performance in the 1st, 2nd, and 3rd step discrimination, respectively. The corresponding confusion matrices for pairwise discrimination in the internal validation dataset were presented in Fig. 6E-G, with diagnostic accuracies of 92.3%, 94.7%, and 98.4% for each well-trained model. Impressively, the established model exhibited robust performance for PCa screening, with a sensitivity of 98.6% and specificity of 82.6% in the internal validation cohort (Fig. 6F). ROC analysis comparing PCa and BPH discrimination revealed AUC values of 0.9836 (metabolic model), 0.6757 (tPSA), and 0.6540 (fPSA/tPSA) in the discovery cohort (Fig. 6H). In addition, Fig. 6I, J displayed the detection rates of PCa group at different specificities in the discovery and internal validation cohorts, respectively. Compared to tPSA and fPSA/tPSA, the metabolic model significantly improved the sensitivity in PCa patients to above 90%. Taking PSA level, Gleason score, International Society of Urological Pathology (ISUP) stage, and tumor metastasis into consideration, this model still displayed acceptable sensitivity of over 90% in all groups, indicating a broad application scenario of this metabolic model (Figure S16).

### Stepwise diagnostic model for prostate cancer

**Fig. 7:**
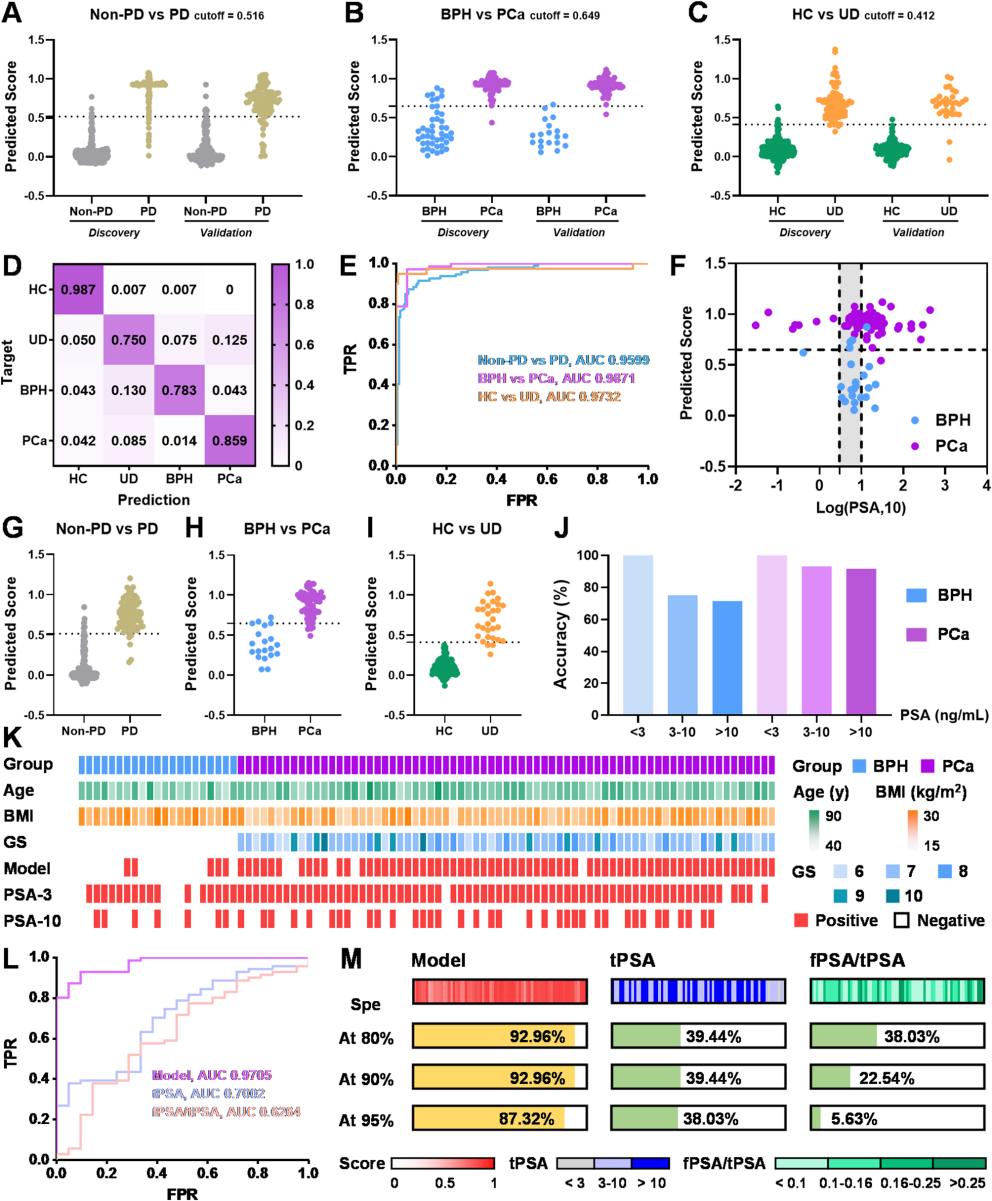
Stepwise diagnostic model promote effective management of urinary diseases. (**A-C**) Predicted scores for the first-layer PD diagnosis, second-layer PCa discrimination and third-layer UD recognition respectively. (**D**) Heatmap displays the ratios of predicted cases to true cases in the verification dataset. (**E**) Receiver operating curves for the stepwise prediction in the internal validation cohort. (**F**) 2D coordinate plot visualizes diagnostic results for each sample based on PSA levels and established model. (**G-I**) Predicted scores of each step discrimination in the independent validation cohort. (**J**) Accuracy of BPH and PCa patients with different PSA levels. (**K**) Baseline information and diagnostic results of BPH and PCa subjects in the external validation cohort. (**L**) Receiver operating curves and (**M**) detection rates of PCa group at different specificities in the independent validation cohort

In general, significant peak signals were extracted and input for step-by-step identification following rapid MS detection. Notably, SVM, SVM, and LASSO regression algorithms were selected for the 1st, 2nd, and 3rd step discrimination. The predictive performance was evaluated in both the discovery cohort (*n* = 567) and internal validation cohort (*n* = 284), with detailed score distributions presented in Fig. 7A-C. As shown in Fig. 7D, subjects in the internal validation cohort were classified with an accuracy of 98.7% for HC, 75.0% for UD, 78.3% for BPH and 85.9% for PCa. In addition, AUC values from the ROC curves that differentiate Non-PD from PD, BPH from PCa, and HC from UD were 0.9599, 0.9871, and 0.9732, showing satisfactory diagnostic performance (Fig. 7E, Figure S17A). As shown in Fig. 7F, the established model significantly improved diagnostic sensitivity for PCa patients in the gray area (3 < tPSA < 10 ng/mL). To confirm the reliability of our model, an external validation cohort was independently collected and analyzed, including 150 HC, 40 UD, 21 BPH and 71 PCa subjects (Table S3). The predicted scores, corresponding confusion matrices and ROC curves of each step discrimination were pictured in Fig. 7G-I and Figure S17B-E. Overall, subjects in the external validation cohort were classified with an accuracy of 99.3% for HC, 67.5% for UD, 76.2% for BPH and 87.3% for PCa (Figure S17F). In the second prediction step, the detection rates of BPH and PCa patients with different PSA levels were summarized in Fig. 7J. This model can successfully identify 71.43% of BPH volunteers with PSA > 10 ng/mL, while PCa patients with different PSA levels had a diagnostic accuracy of more than 91.67%. Furthermore, Fig. 7K visualized the baseline information and diagnostic results for each PD sample in the external validation set. Clearly, nearly half of BPH patients showed false-positive results when we set a diagnostic threshold of PSA > 3 ng/mL. When the diagnostic threshold was set at 10 ng/mL, the PSA index was less sensitive to PCa patients. As shown in Fig. 7L, AUC values from the ROC curves were 0.9705 (model), 0.7002 (tPSA), and 0.6264 (fPSA/tPSA), respectively. Figure 7M summarized the detection rates of PCa patients in the external validation cohort at the specificity of 80%, 90% and 95%. When the specificity was set at 95%, the sensitivity of our model, tPSA, and fPSA/tPSA for PCa diagnosis was 87.32%, 38.03%, and 5.63%, respectively. For samples with abnormal PSA levels (PSA > 3 ng/mL) in the external validation set, the negative and positive predictive values were 73.33% and 92.31%, which enabled 73.33% of BPH samples to avoid excessive biopsy. Of note, current clinical guidelines clearly describe the goal of biomarker testing as the detection of clinically significant cancer [22] (Gleason score ≥ 7). Thus, we discussed the results of distinguishing benign from GS7-10 as the ultimate clinical goal. As shown in Figure S18A-D, the diagnostic sensitivity of the constructed model for significant PCa patients in the discovery, internal validation, and external validation cohorts was above 90%. When thresholds were set at PSA > 10 ng/mL, 58.33% of significant PCa patients in the external validation cohort were missed, respectively; Surprisingly, the established technique was able to detect 93.33% of clinically significant prostate cancer. Second, we found that more than 95% of significant PCa patients in the gray area (3 < tPSA < 10 ng/mL) were successfully identified (Figure S18E), demonstrating the promising prospect of high-throughput urine metabolomics in clinical diagnosis.

## Discussion

Metabolomics has exhibited great potential in discovering disease [29]. We hypothesized that urine metabolomics may shed light on monitoring the molecular changes during prostatic carcinogenesis and provide a new dimension to PCa screening in large populations.

In this work, untargeted metabolomics was employed to explore associations of metabolites with PCa phenotype. Forty-eight metabolites were verified to be dysregulated in the PCa and fourteen metabolites were differentially expressed across all PD groups. The levels of proline, imidazolepropionic acid, caprylic acid, imidazolelactic acid, N-Acetylproline, L-Dihydroorotic acid and N-Acetylmethionine displayed a constant upward trend, whereas GABA, malonic acid, allysine, glutamic acid, methionine, N-Acetylvaline and phthalic acid exhibited a markedly downward trend. Impressively, abnormal fluctuations of most amino acids and secondary metabolites in PCa patients have been observed in numerous studies [16, 17, 30–32]which may be the consequence of protein malnutrition and the increase in amino acid demand. In the human body, L-Dihydroorotic acid can be converted into orotic acid, which serves as an intermediate in pyrimidine biosynthesis [33]. Additionally, the upregulation of caprylic acid and downregulation in malonic acid indicate potential fatty acid synthesis disruption in prostate cells [16, 34]. Besides known disease-related biomarkers, this study found changes in phthalic acid that could link to cancer development.

Amino acid and nucleotide metabolisms were globally disrupted in PCa. Imbalanced amino acid metabolism acts as a metabolic regulator supporting cancer cell growth [35]. Tumor cell proliferation, chemoresistance, and immune evasion heavily depend on enhanced nucleotide metabolism [36]. TCGA transcriptomic data confirmed urine metabolomics results, showing disruptions in amino acid and nucleotide metabolisms, with 23 metabolite-related genes differentially expressed in prostate cancer (PCa) versus normal tissues. The biological relevance of *AOX1*, *PON3*, *CBS* and *ASPA* was elucidated in PCa. CBS activity is regulated by S-adenosine methionine (SAM), the primary methyl donor in methionine metabolism [37]. N-acetylmethionine and methionine are involved in the synthesis of SAM [38]. Our study revealed significant decreases in N-acetylmethionine and methionine in PCa urine, indicating a potential SAM deficiency affecting CBS activity. Hence, prostate cancer tissues may upregulate CBS expression to compensate and restore metabolic balance, explaining the observed increase in CBS in these tissues.

In clinical diagnosis, serum PSA level serves as a tumor biomarker for prostate cancer. However, there is still a lack of a definitive PSA range showing considerable sensitivity and specificity values [39]. Previous clinical studies demonstrated that PSA had a detection rate of 21.9% for PCa diagnosis when the cut-off value was set as 3 ng/mL [40]. PCA3 has emerged as a valuable PCa marker, with a multicenter study reporting 64% sensitivity and 76% specificity [41]. Detecting metabolic signatures linked to prostatic carcinogenesis could provide a promising diagnostic approach for PCa. In clinical implementation, integrating the metabolic signature-based diagnostic model with fPSA measurements optimizes the management of patients with gray-zone fPSA (3–10 ng/mL), achieving a 25.0% reduction in unnecessary biopsies at the cost of a 6.9% PCa false-negative rate when applying the predefined cutoff (0.649) derived from discovery and internal validation cohorts. While the initial procurement cost of LDI-MS systems is substantial, their capacity for high-throughput multi-analyte analysis may yield superior cost-effectiveness relative to conventional single-analyte testing methods. Benefiting from streamlined sample preparation and second-level mass spectrometry scanning, this approach achieves high-throughput detection of 96 urine samples within a 40-minute turnaround time. Through automation, standardization, and AI-assisted data analysis, LDI-MS has the potential to become a vital tool in clinical laboratories.

In summary, we introduced an efficient, high-throughput PCa screening method with good specificity using LDI-MS. It correctly identified 87.3% of PCa patients in an external validation set, validating its potential as a diagnostic tool. Moreover, the stepwise model successfully identified 99.3% of HC, 67.5% of UD, and 76.2% of BPH patients. In the future, this metabolic signature-based diagnostic model may serve as a triage test to reduce unnecessary biopsies in high-risk populations with elevated PSA levels.

Despite promising outcomes, our study has limitations: (1) The cross-sectional nature of this study precludes establishing a causal relationship between metabolic dysregulation and PCa pathogenesis; therefore, longitudinal follow-up studies or functional investigations are warranted to elucidate these associations. (2) To address potential population bias and limited generalizability due to the single-center design, future validation across multiple medical centers is required to demonstrate the widespread applicability of this technology.

## Electronic supplementary material

Below is the link to the electronic supplementary material.

Supplementary Material 1

## Data Availability

Data can obtain with the permission of the corresponding author.

## Abbreviations

PCa: Prostate cancer
BPH: Benign prostatic hyperplasia
LDI-MS: Laser desorption/ionization mass spectrometry
MALDI-TOF MS: Matrix-assisted laser desorption/ionization time-of-flight mass spectrometry
OPLS-DA: Orthogonal partial least squares-discrimination analysis
PSA: Prostate specific antigen
KEGG: Kyoto Encyclopedia of Genes and Genomes
