# Supplementary material for: High-throughput LDI MS technology decodes the distinct metabolic landscape of prostate cancer in a large-scale cohort
Source: Biomark Res. 2025 Jul 9;13:94. doi: 10.1186/s40364-025-00804-z (PMC12239324; doi:10.1186/s40364-025-00804-z)
Supplement: Supplementary file 1 — Supplementary Material 1 [file 40364_2025_804_MOESM1_ESM.docx]

**Table of Supporting Information Contents:**

***1. Supplementary Experimental Procedures***

***1.1*** Detailed experimental parameters of UPLC-MS analysis

***1.2*** Cell lines and culture

***1.3*** Tissue collection and preparation

***1.4*** Machine learning

***1.5*** Detailed procedure of transcriptome analysis

***2. Supplementary Figures***

***Figure S1*** Distribution of propensity scores in the disease and control groups.

***Figure S2*** Reproducibility of urine detection within the same batch or between different batches.

***Figure S3*** The OPLS-DA scores plot for QC sample and clinical samples involved in this project.

***Figure S4*** MS spectra and MS/MS fragment profiles of urinary metabolites and commercial standard reagents.

***Figure S5*** Intersection analysis of enriched pathways disturbed in the three groups.

***Figure S6*** Common disturbed metabolic pathways in different phenotypes.

***Figure S7*** Volcano plot showing the differential expressed genes between PCa tumors and non-tumor adjacent normal tissues.

***Figure S8*** KEGG pathway enrichment analysis of differential genes and dysregulated metabolites.

***Figure S9*** Compound-reaction-enzyme-gene network built by *MetScape* on the basis of PCa-related metabolites.

***Figure S10*** The expression of candidate genes in prostate cancer from open-source TCGA database.

***Figure S11*** Survival analysis of candidate genes expressions in PCa patients.

***Figure S12*** Correlation between the levels of candidate gene expression and nodal metastasis status in PCa patients.

***Figure S13*** Correlation between the levels of candidate gene expression and patient’s Gleason score.

***Figure S14*** The expression of candidate genes in prostate cancer from open-source Asian datasets.

***Figure S15*** Perturbations of metabolite-related genes in PCa compared to BPH.

***Figure S16*** Predicted score distribution of PCa patients in different groups sorted by PSA level, Gleason score, ISUP stage, and tumor metastasis.

***Figure S17*** Discrimination results for discovery and external test sets with the built model.

***Figure S18*** Discrimination results for significant PCa patients.

***3. Supplementary Tables***

***Table S1.*** Clinical characteristics of the subjects in the discovery cohort.

***Table S2.*** Clinical characteristics of the subjects in the internal validation cohort.

***Table S3.*** Clinical characteristics of the subjects in the independent validation cohort.

***Table S4.*** Clinical characteristics of the subjects for RT-PCR and Western Blot analysis.

***Table S5.*** Summary of feature peaks that could be used to distinguish PCa patients from healthy controls.

***Table S6.*** Summary of feature peaks that could be used to distinguish BPH patients from healthy controls.

***Table S7.*** Summary of feature peaks that could be used to distinguish PCa patients from BPH subjects.

***Table S8.*** LC-MS/MS identification of potential feature peaks for discrimination of patients with prostate disease.

***Table S9.*** MALDI-TOF/TOF tandem mass spectrometry identification of urine metabolites.

***1. Supplementary Experimental Procedures***

***1.1*** Detailed experimental parameters of UPLC-MS analysis

Here, the detailed experimental parameters of UPLC-MS analysis of urine samples were summarized. Frozen urine was thawed on ice before the UPLC-MS analysis. Subsequently, the urine sample (200 μL) was mixed with acetonitrile (200 μL) to precipitate proteins. After vortexing for 60 s, incubating for 10 min and storing at -20°C overnight, the mixtures were centrifuged at 4000 g for 20 min at 4℃. The supernatant was filtered through syringe filters (0.22 μm) before UPLC-MS analysis. RPLC separation was performed on an ACQUITY UPLC T3 column (2.1×100 mm, 1.8 μm, Waters, UK). The column was maintained at 35℃. In RPLC mode, gradient elution was performed with the following solvent system: (A) 0.1% formic acid-water, (B) ACN with 0.1% formic acid. The gradient elution conditions were as follows with a flow rate of 0.4 mL/min: 5% solvent B for 0-0.5 min; 5-100% solvent B for 0.5-7 min; 100% solvent B for 7-8 min; 100-5% solvent B for 8-8.1min; and 5% solvent B for 8.1-10min. Mass spectrometry experiments were performed on a Triple TOF 5600 Plus high-resolution tandem mass spectrometer (SCIEX, Warrington, UK) with both positive and negative ion modes. The ion spray floating voltage was set at 5kV for the positive-ion mode and ‐4.5kV for the negative-ion mode. The MS data were acquired in the IDA mode and the m/z range was set at 60-1200 Da.

***1.2*** Cell lines and culture

RWPE-1 cells were cultured in [McCoy's 5A](http://www.baidu.com/link?url=0ws8yst0HDrSaGhwDv8qKkPBuo4oOgIzZNGwEvkBgjQRDOlf-s6VY-rSbTAtkk_hOUc-2MStrzQuth5pISPJ7zfcfqmg1-cqdQTjCHqTflu) medium (Servicebio), PC3, C4-2 and C4-2B cells were cultured in RPMI-1640 medium (Thermo Fisher Scientific), and DU145 cells were cultured in MEM (Pricella). The cells mentioned above were supplemented with 10% FBS (Gibico), 1% penicillin (Biosharp) and 1% streptomycin (Biosharp) (penicillin-streptomycin) at 37°C under 5% CO_2_. WPMY-1 were cultured in DMEM (Thermo Fisher Scientific) supplemented with 5% FBS (Gibico), 1% penicillin (Biosharp) and 1% streptomycin (Biosharp) (penicillin-streptomycin) at 37°C under 5% CO_2_. All cells are negative for mycoplasma infection.

***1.3*** Tissue collection and preparation

Prostate cancer and paired adjacent tissues were collected from patients undergoing radical prostatectomy at Sir Run Run Shaw Hospital, Zhejiang University School of Medicine. The clinical characteristics of all participants in tissue collection project are shown in Table S4.

***1.4*** Machine learning

The retrospective cohort collected from June 1^st^, 2021 to December 31^st^, 2022 was employed for model establishment and parameter optimization, and the retrospective cohort was divided into training set and internal validation set at a ratio of 2:1. The independent cohort collected from January 1^st^, 2023 to April 30^th^, 2023 was employed for model external validation. Discriminant models were constructed by the in-built “Regression learner” and “Classification learner” modules in MATLAB software via cross-validation (10-folds). Seven machine learning algorithms were investigated in this work, including decision trees (DT), nearest neighbor classifier (KNN), naïve Bayesian classifier (NB), LASSO regression, linear discriminant analysis (LDA), logistic regression (Logi), and support vector machine (SVM). Kappa statistic, accuracy, F-measure, precision, recall and the area under the curve (AUC) were utilized to evaluate the classification performance of different machine learning models. Sensitivity, specificity and AUC were selected as diagnostic indicators to evaluate the classification results of the prediction model in the training, internal validation and external validation set.

***1.5*** Detailed procedure of transcriptome analysis

The transcriptomics profiles of PCa tumor and adjacent normal tissues were downloaded from open-source TCGA dataset. Statistical analysis was performed using R 3.5.2 software and the differentially expressed genes were screened out by the R edgeR package. Statistical significance was defined as FDR < 0.05 and |log_2_FC| > 1. Gene ontology and KEGG pathway enrichment analysis were performed by online KOBAS database (http://kobas.cbi.pku.edu.cn/kobas3). Kaplan–Meier survival analysis was completed with GEPIA (http://gepia.cancer-pku.cn). Furthermore, correlation analysis of clinical parameters with the mRNA expression of enzyme genes was investigated by UALCAN website (http://ualcan.path.uab.edu).

***2.*** ***Supplementary Figures***

**
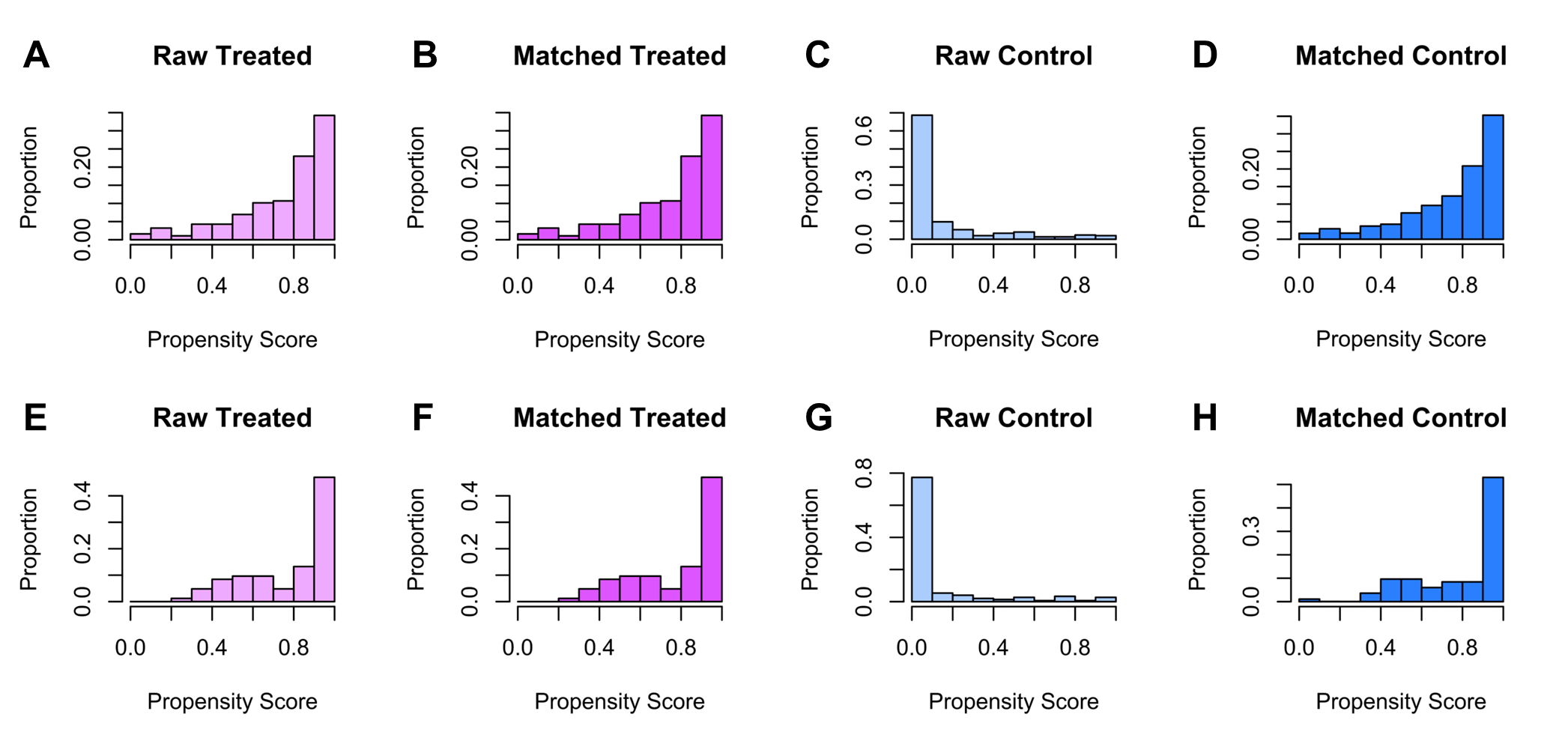
**

***Figure S1*** Distribution of propensity scores in the disease and control groups. (A)(B) Disease group in the retrospective cohort. (C)(D) Healthy control group in the retrospective cohort. (E)(F) Disease group in the external validation cohort. (G)(H) Healthy control group in the external validation cohort.

***Figure S2*** Reproducibility of urine detection within the same batch or between different batches. (A) Intra-batch and (B) inter-batch stability for metabolite profiles in QC sample, respectively.


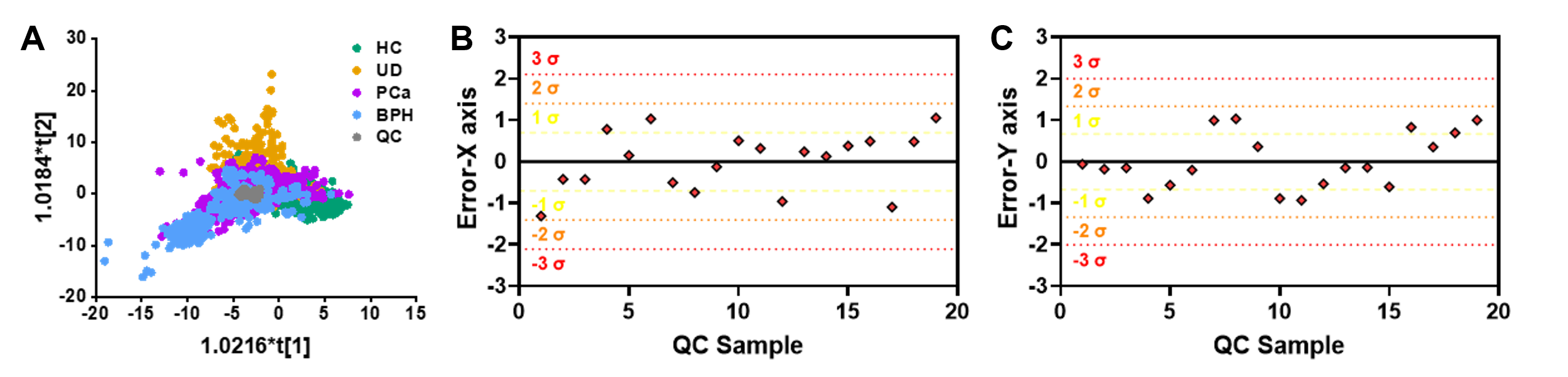


***Figure S3*** The OPLS-DA scores plot for QC sample and clinical samples involved in this project. (A) OPLS-DA scores plot. (B)(C) Line plots of QC samples for LDI‐MS analysis generated by OPLS-DA. (B) Distribution of the X-axis error. (C) Distribution of the X-axis error.

**
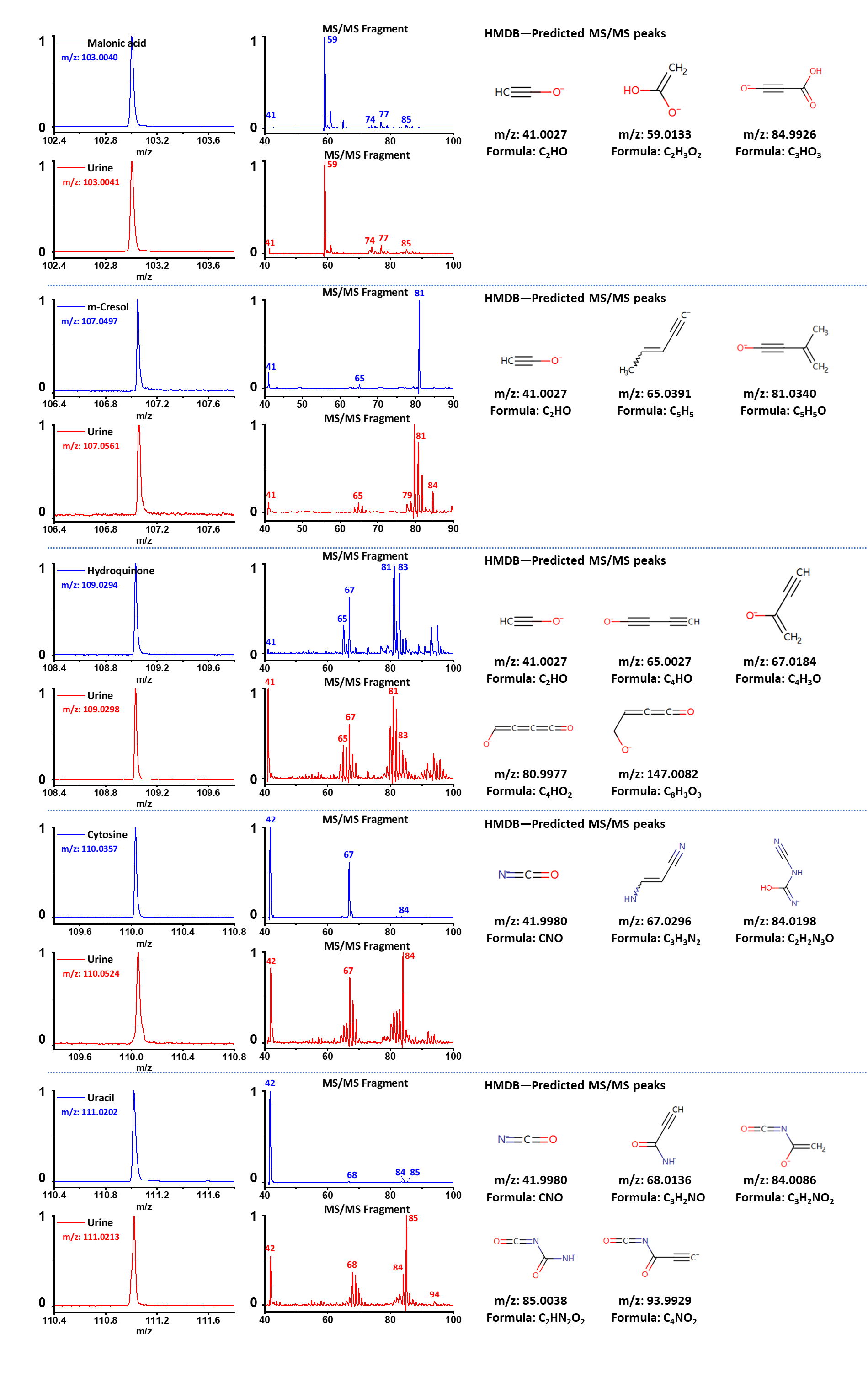

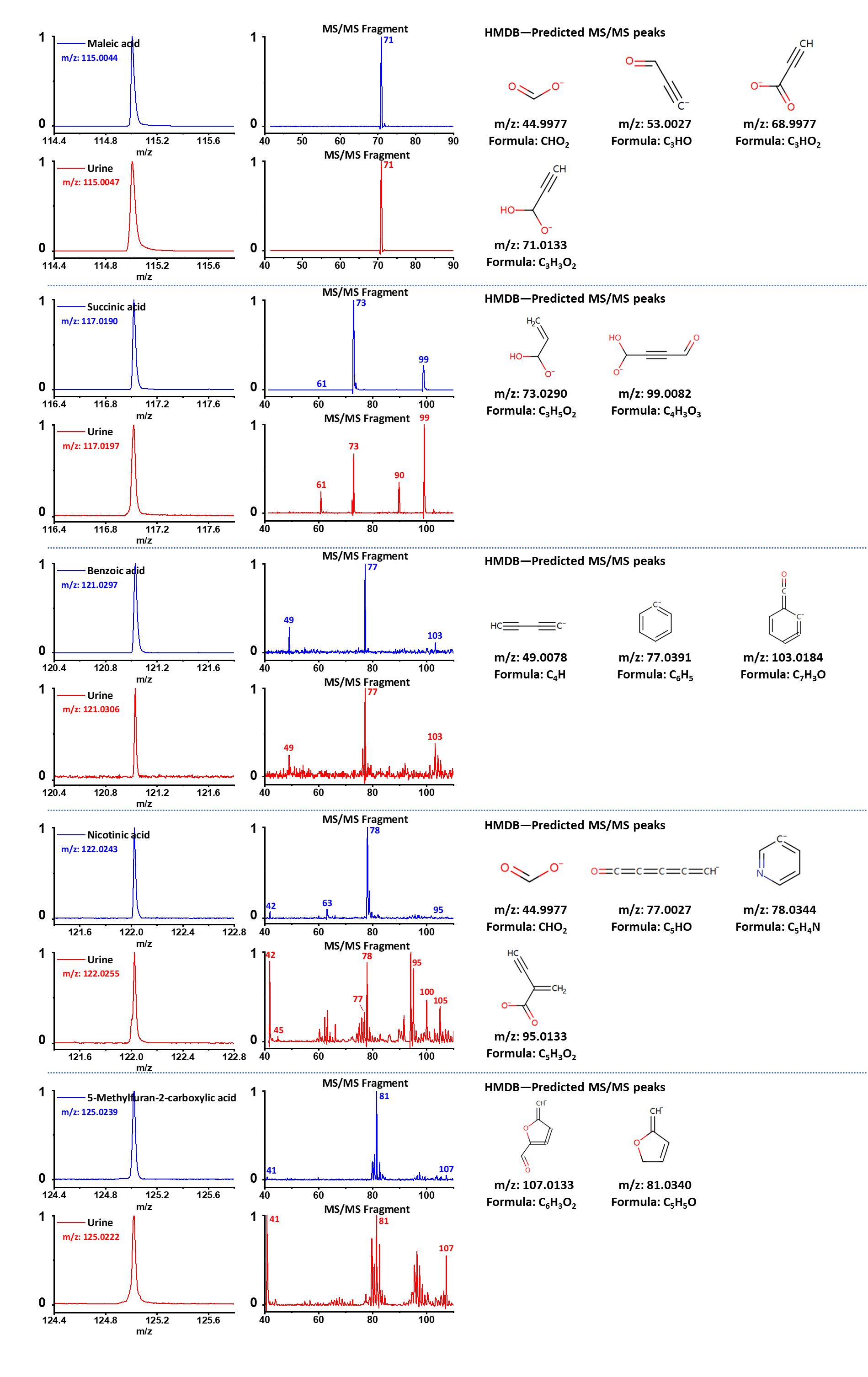

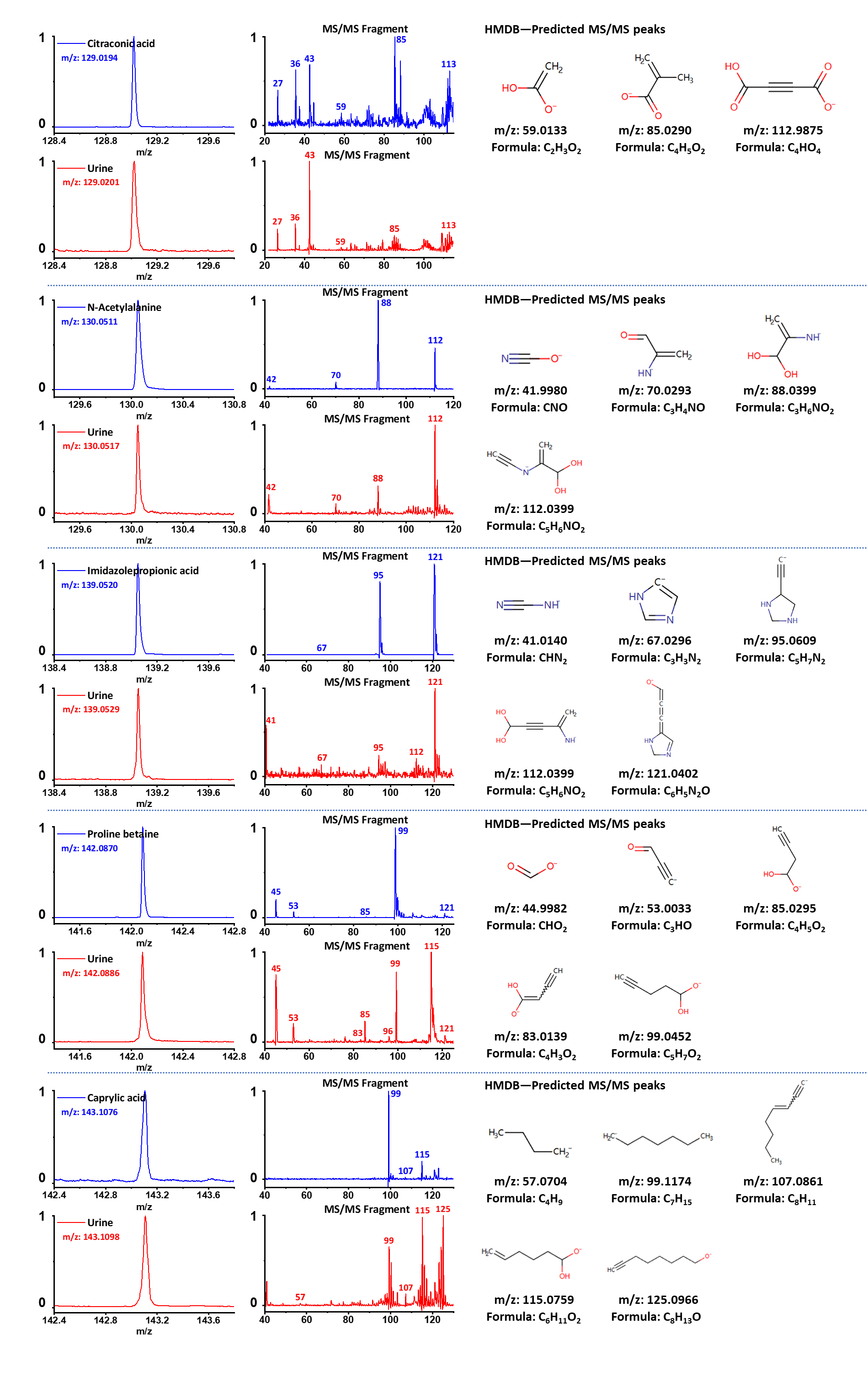

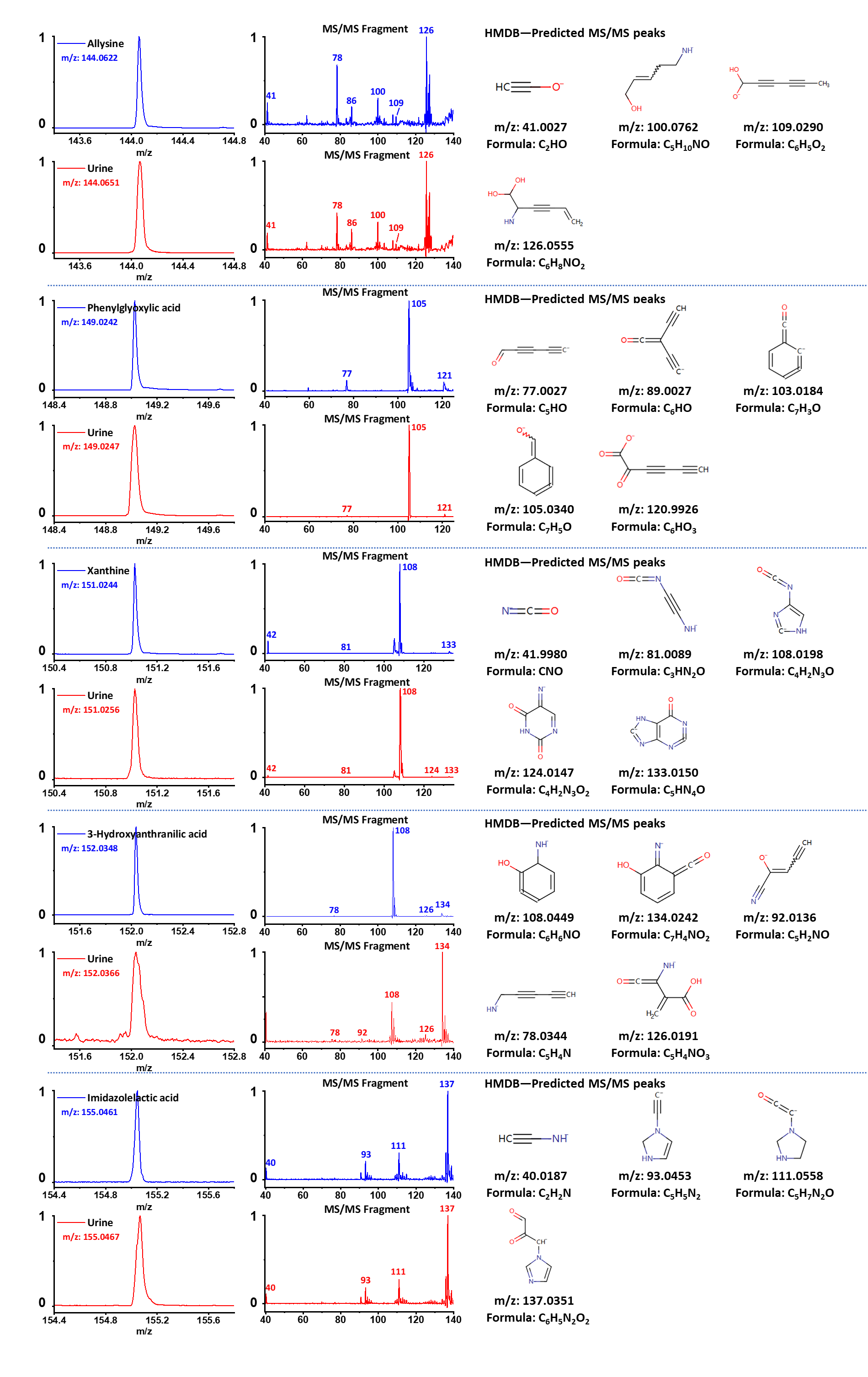

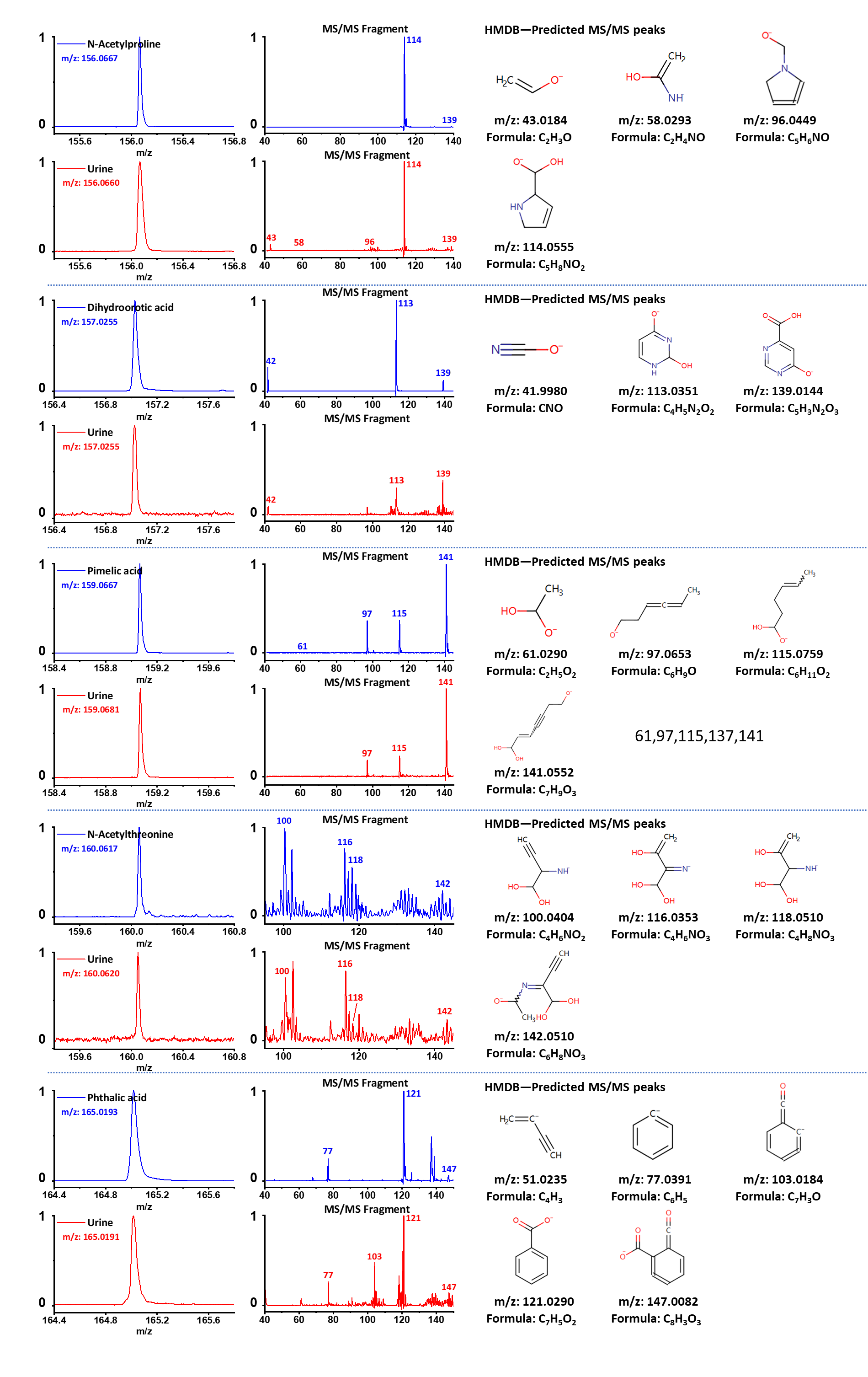

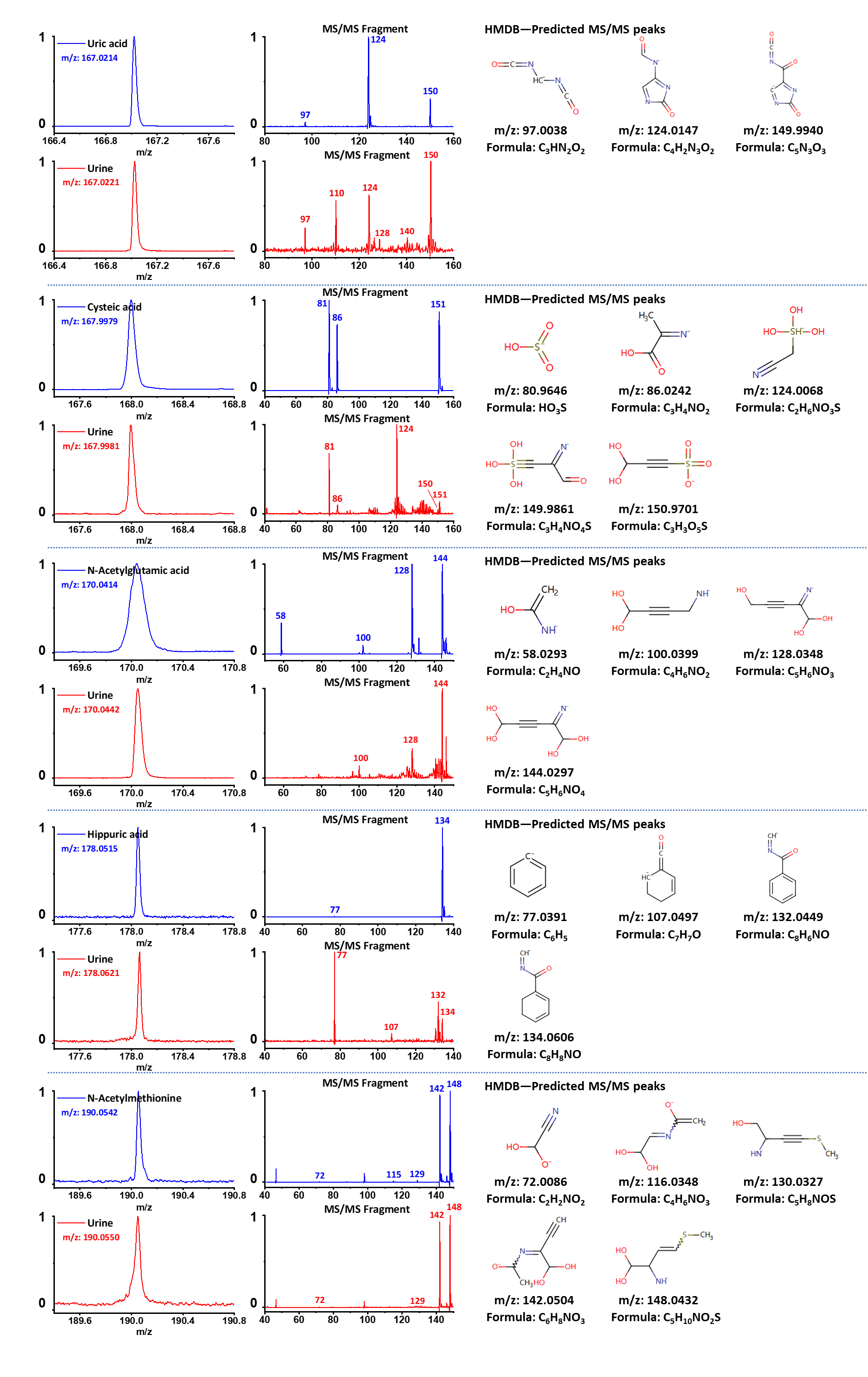

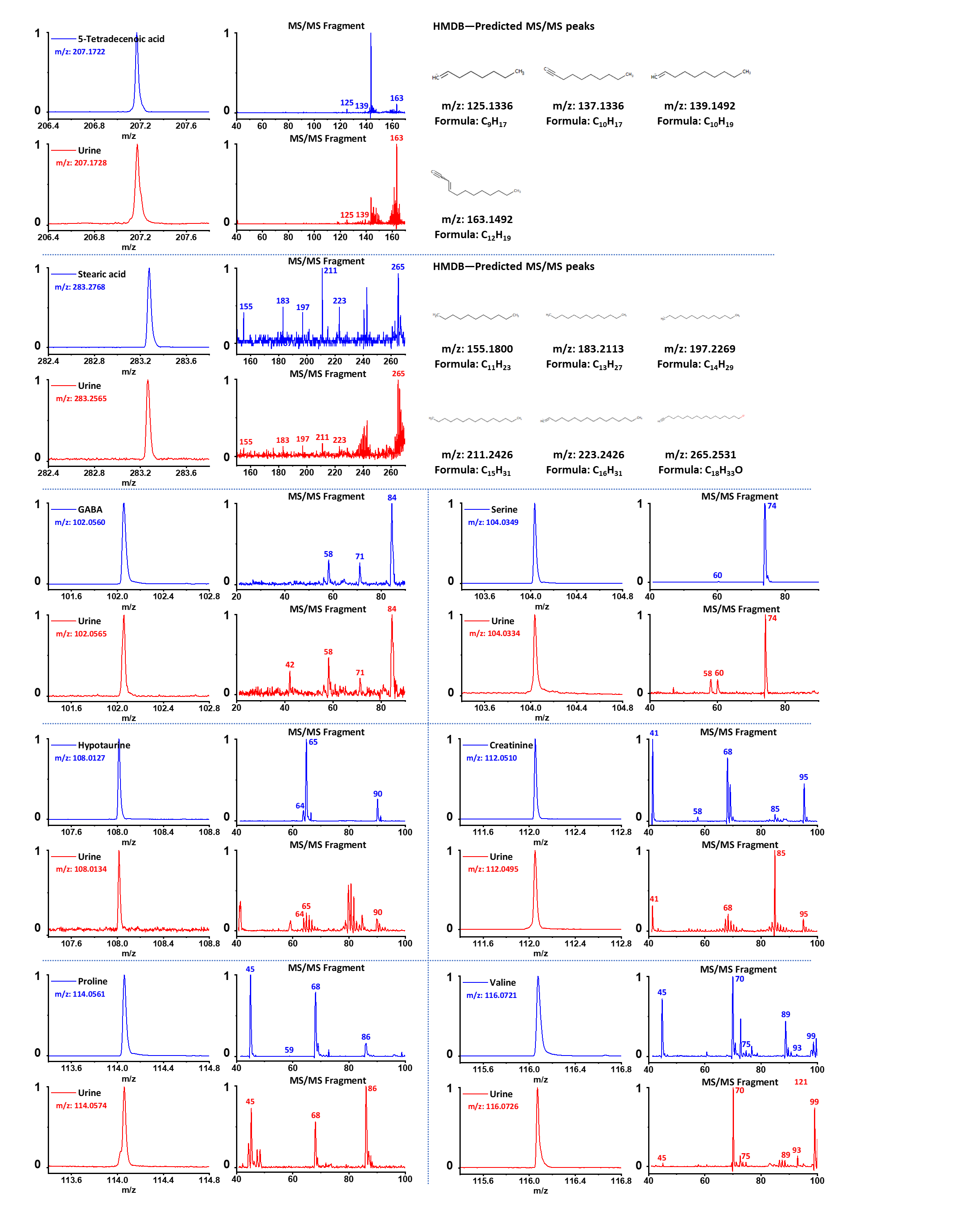

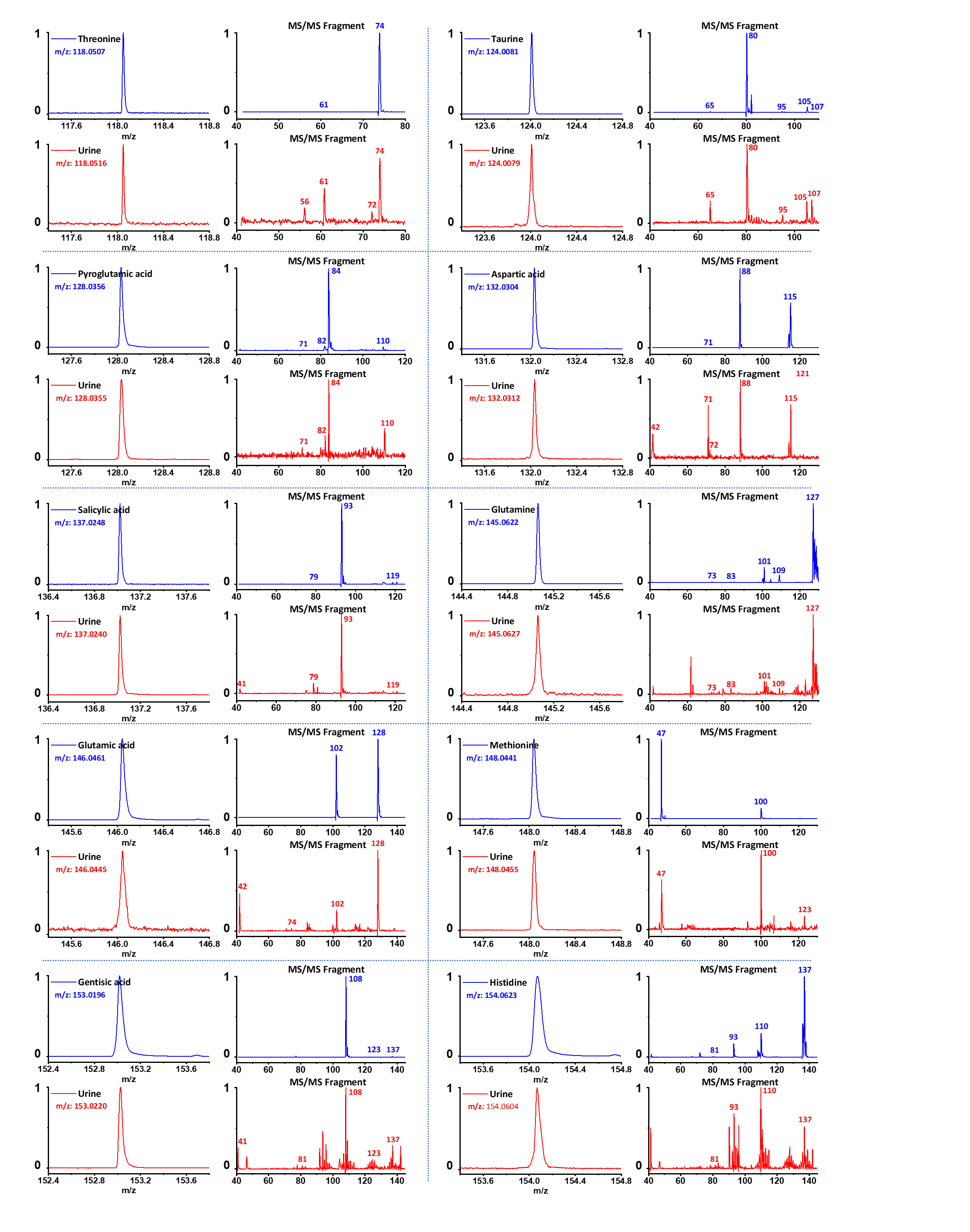

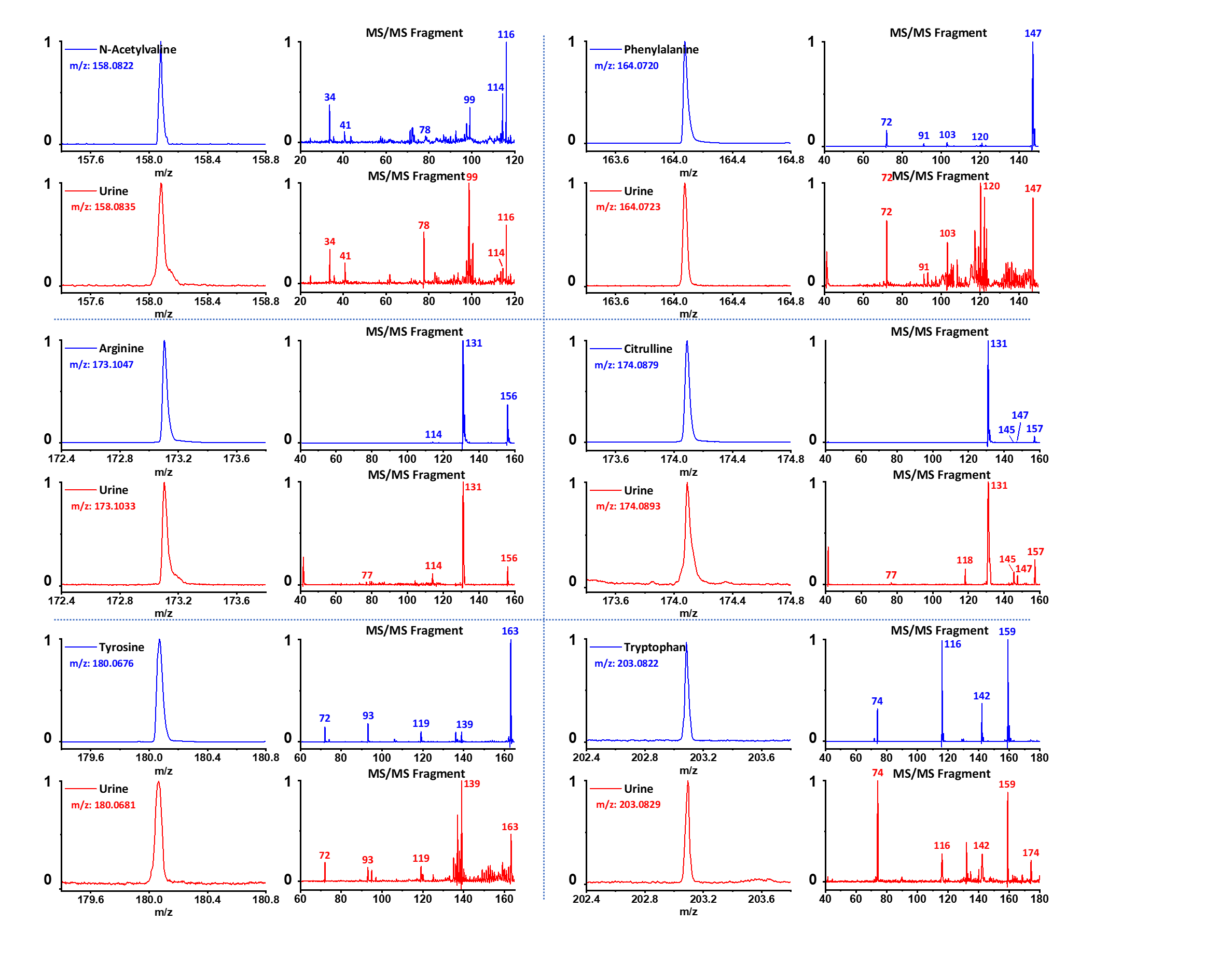
**

***Figure S4*** MS spectra and MS/MS fragment profiles of urinary metabolites and commercial standard reagents. Note: Predicted fragment peaks for several metabolites are not available in the HMDB database.


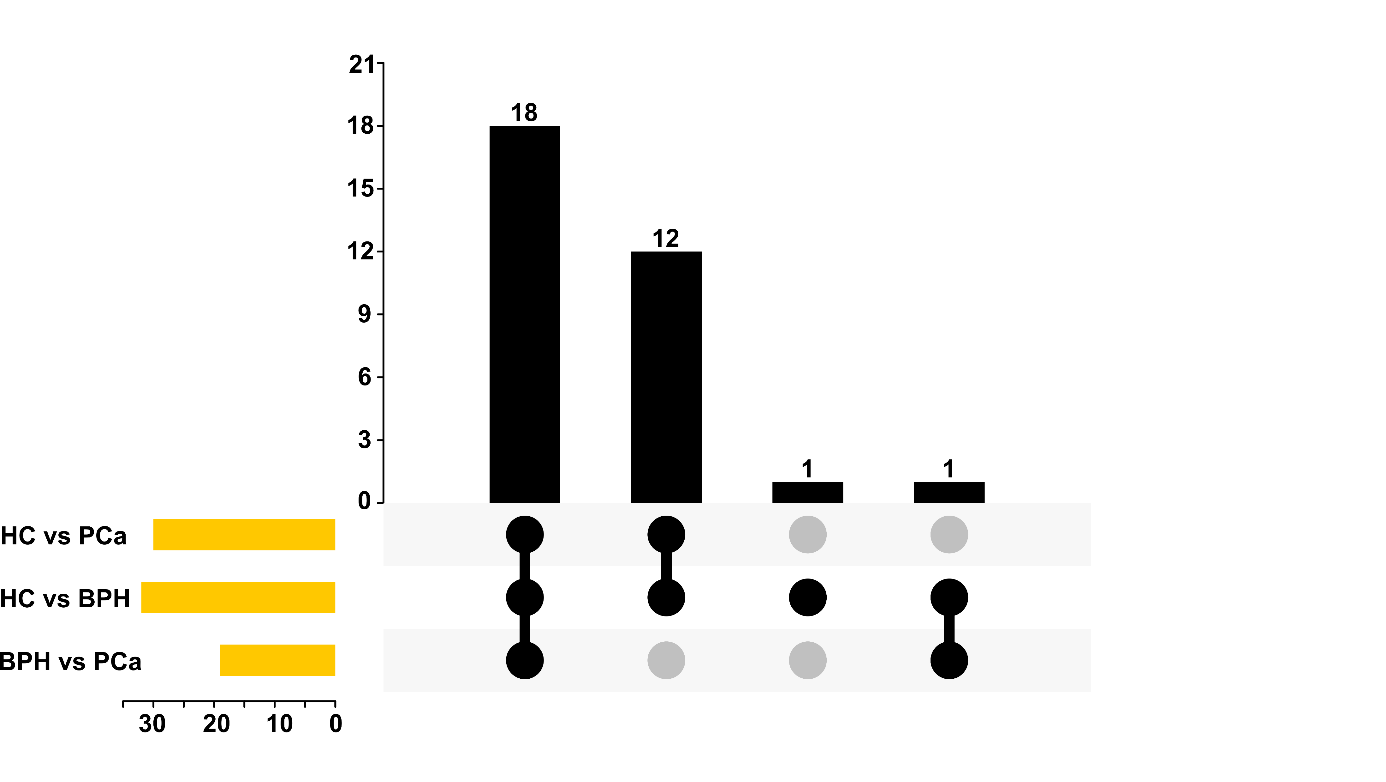


***Figure S5*** Intersection analysis of enriched pathways disturbed in the three groups. The bar chart at the bottom left represents the number of processes included in each group. The black bar chart above represents the number of common processes in each intersection.

***Figure S6*** Common disturbed metabolic pathways in different phenotypes. The circle colors represent JG scores of certain disturbed pathways between two groups. The circle size is proportional to the log10(p) value. In the left ribbon, purple, blue and yellow represent pairwise analysis between BPH and PCa, HC and BPH, HC and PCa, respectively. Map_null_1 refers to D-Glutamine and D-glutamate metabolism and map_null_2 refers to porphyrin and chlorophyll metabolism, which do not match the corresponding numbers in the KEGG database.


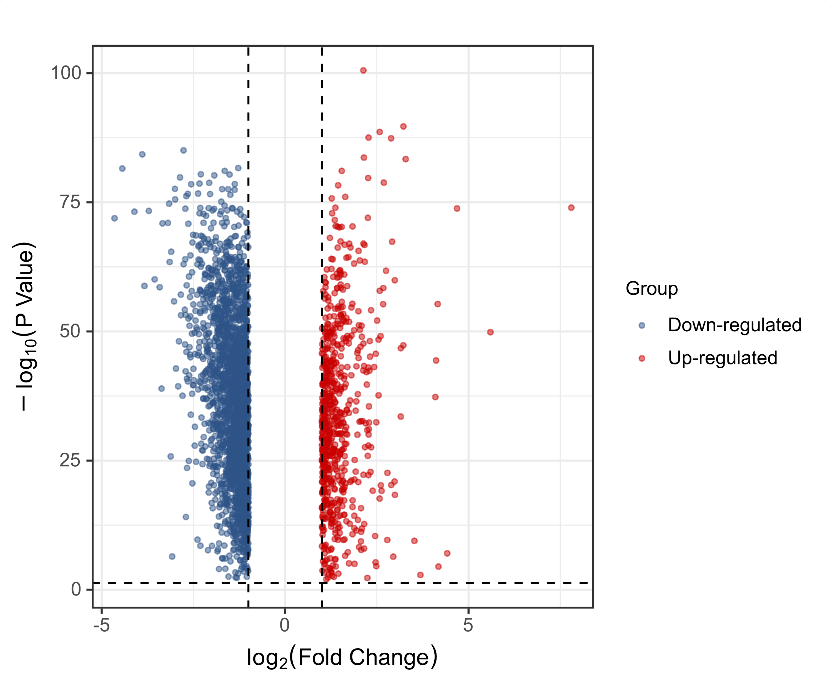


***Figure S7*** Volcano plot showing the differential expressed genes between PCa tumors and non-tumor adjacent normal tissues. Notably, p value was adjusted by Benjamini-Hochberg method.

***Figure S8*** KEGG pathway enrichment analysis of differential genes and dysregulated metabolites.


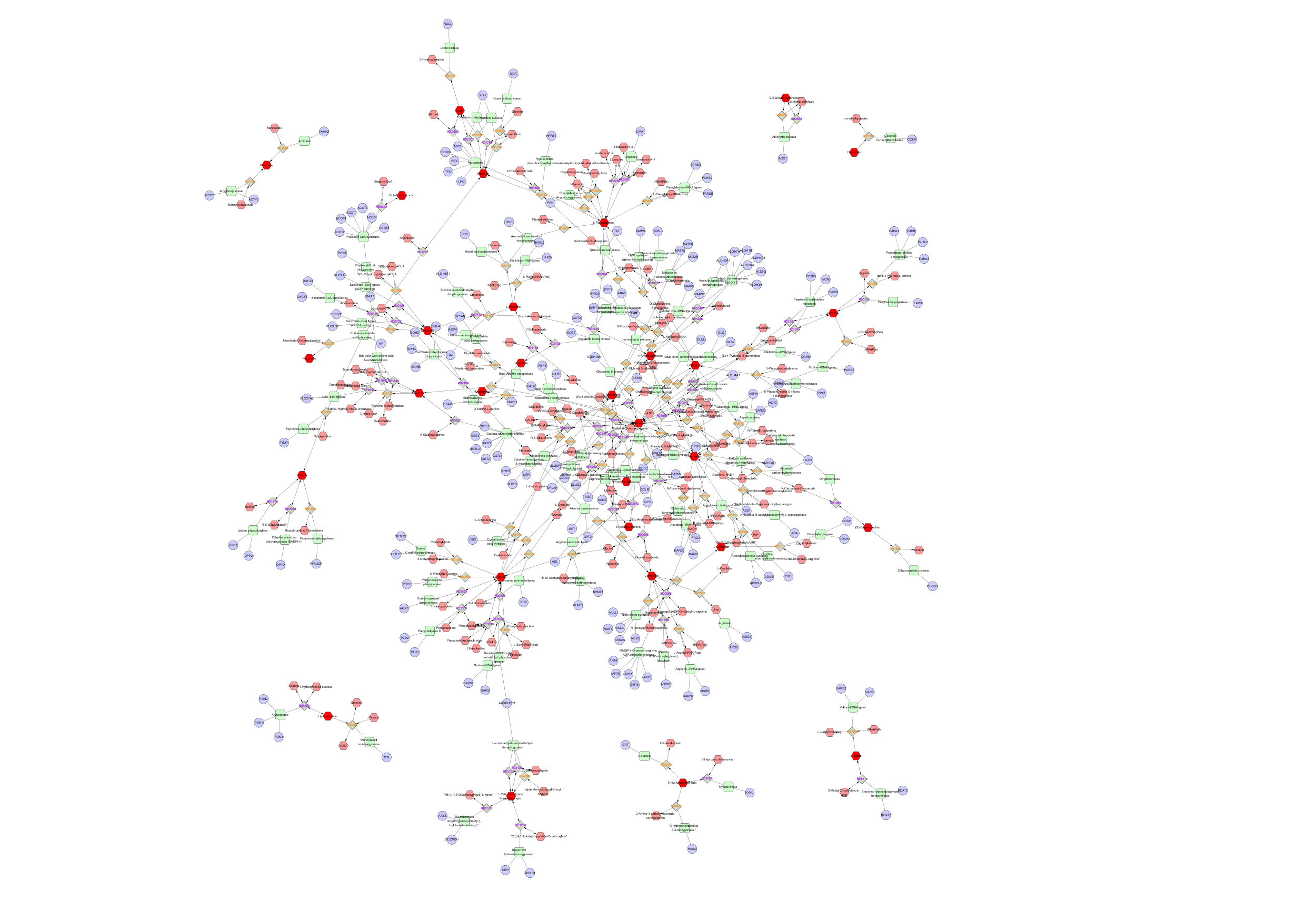


***Figure S9*** Compound-reaction-enzyme-gene network built by *MetScape* on the basis of PCa-related metabolites.

***Figure S10*** The expression of candidate genes in prostate cancer from open-source TCGA database. (A) *AASS*, (B) *AGA*, (C) *ALDH1A3*, (D) *AOX1*, (E) *ASPA*, (F) *BHMT2*, (G) *CBS*, (H) *CSAD*, (I) *DDAH1*, (J) *FTCD*, (K) *GATM*, (L) *GFPT1*, (M) *GFPT2*, (N) *GGT1*, (O) *GPT*, (P) *HAAO*, (Q) *HDC*, (R) *NADSYN1*, (S) *P4HB*, (T) *PON3*, (U) *PYCR1*, (V) *SLC27A2*, and (W) *UPP1*.

***Figure S11*** Survival analysis of candidate genes expressions in PCa patients. (A) Survival map of all differential metabolite-related genes. (B) Kaplan-Meier curve of *NADSYN1*.

***Figure S12*** Correlation between the levels of candidate gene expression and nodal metastasis status in PCa patients.

***Figure S13*** Correlation between the levels of candidate gene expression and patient’s Gleason score.


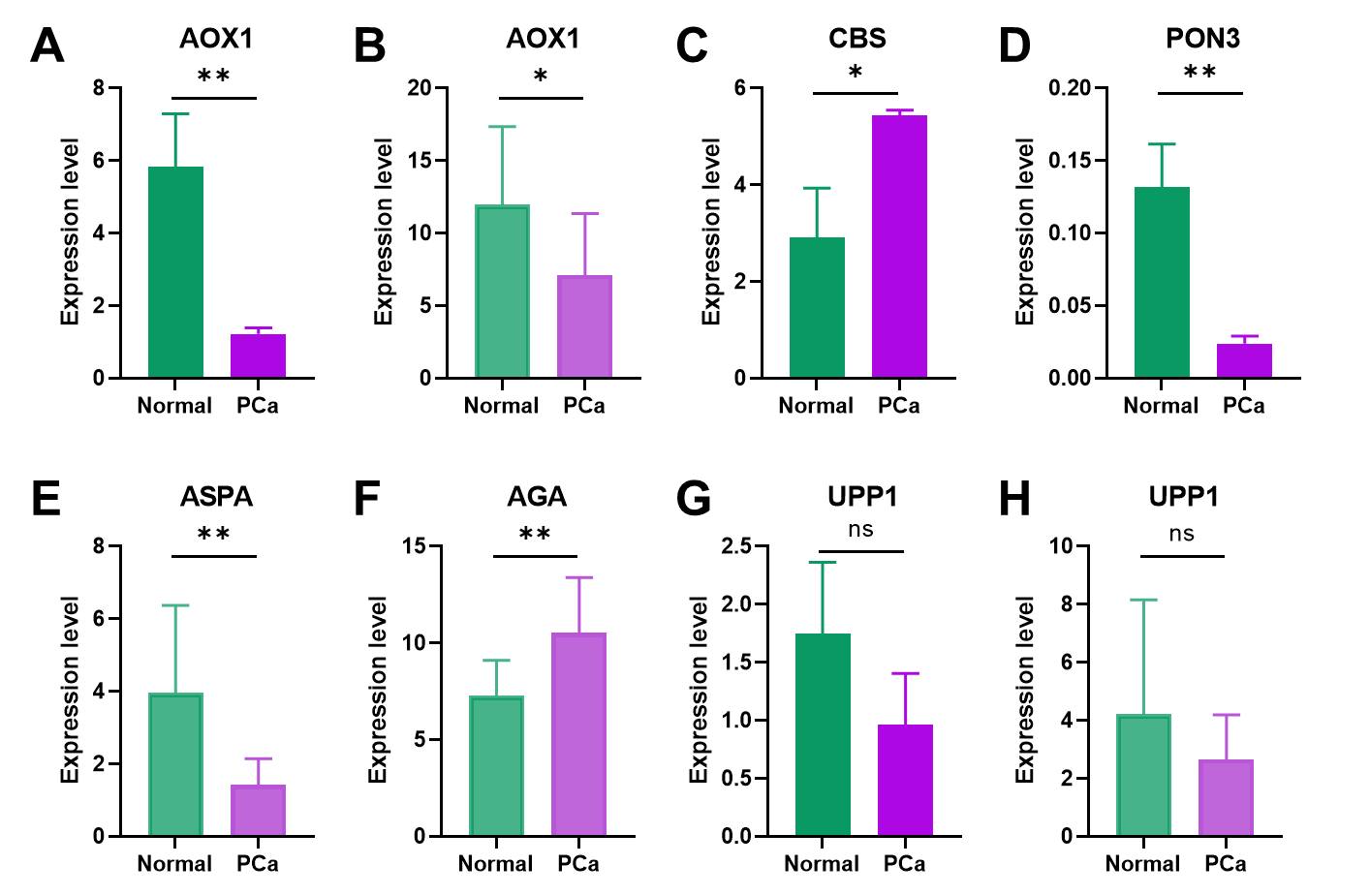


***Figure S14*** The expression of candidate genes in prostate cancer from open-source Asian datasets. (A)(B) The mRNA level of *AOX1* in the (A) GSE179321 and (B) GSE114740 datasets. (C) *CBS* and (D) *PON3* in the GSE179321 dataset. (E) *ASPA* and (F) *AGA* in the GSE114740 dataset. (G)(H) *UPP1* in the GSE179321 and GSE114740 datasets.

***Figure S15*** Perturbations of metabolite-related genes in PCa compared to BPH. (A) Metabolite-reaction-enzyme-gene network based on the differentially expressed metabolites between PCa and BPH groups. (B-D) The expression of differential genes from GSE30994 dataset.

***Figure S16*** Predicted score distribution of PCa patients in different groups sorted by (A) PSA level, (B) Gleason score, (C) ISUP stage, and (D) tumor metastasis.

***Figure S17*** Discrimination results for discovery and external test sets with the built model. (A)(B) Receiver operating curves for the stepwise prediction in the discovery (A) and external validation (B) cohorts, respectively. (C-E) Confusion matrix for the first (C), second (D and third (E) step predictions in the external test sets. (F) Heatmap displays the ratios of predicted cases to true cases in the external test sets.


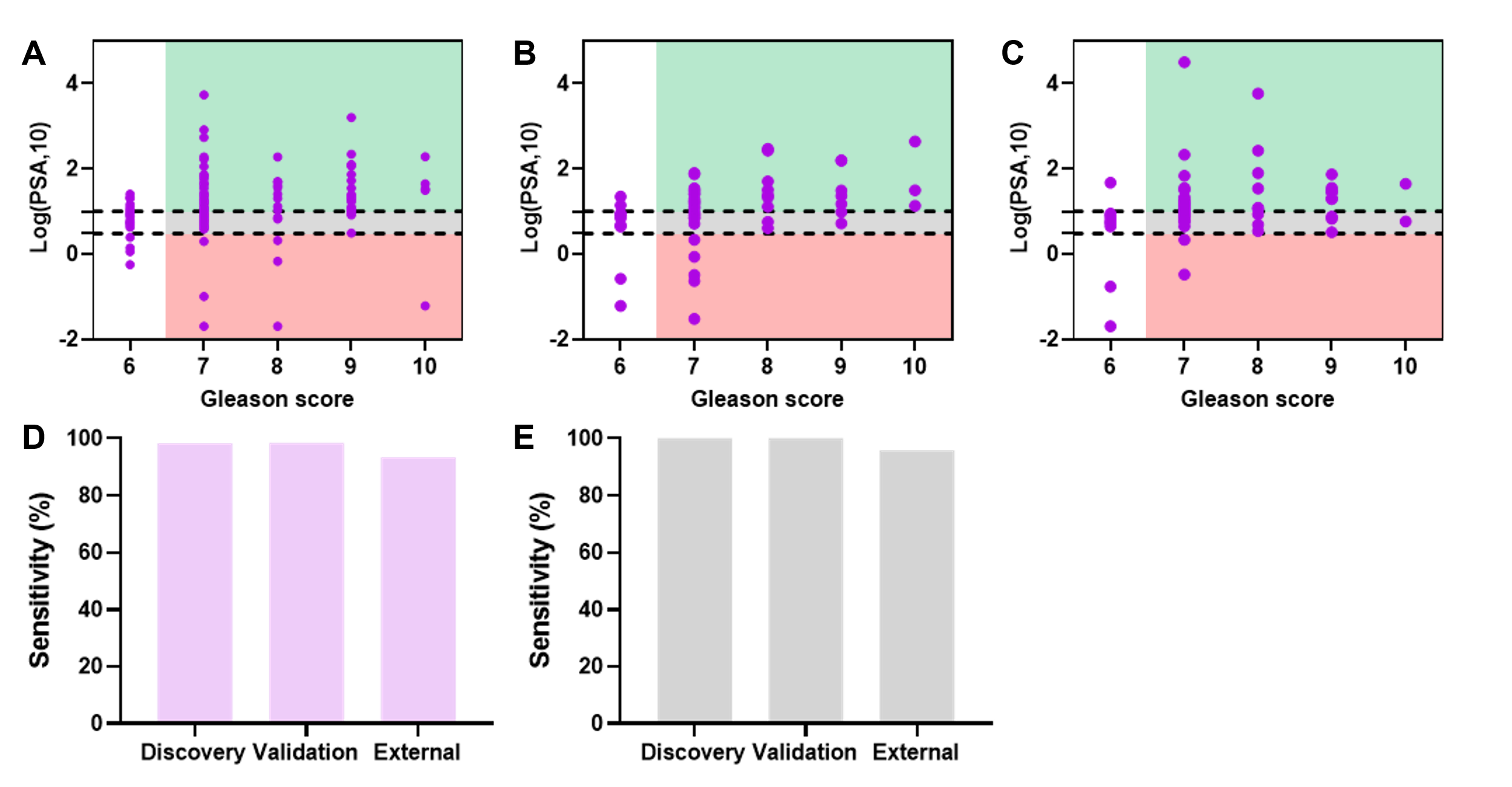


***Figure S18*** Discrimination results for significant PCa patients. (A-C) Gleason score and PSA level (ng/mL) of PCa patients in the discovery, internal validation, and external validation sets, respectively. (D) Diagnostic sensitivity of the constructed model for significant PCa patients (GS ≥ 7). (E) Diagnostic sensitivity of significant PCa patients in the gray area (3 < tPSA < 10 ng/mL).

***3. Supplementary Tables***

***Table S1.*** Clinical characteristics of the subjects in the discovery cohort.

| **Characteristics** | **Discovery set** | | | | **p value** |
| --- | --- | --- | --- | --- | --- |
|  | **HC** | **UD** | **BPH** | **PCa** |  |
| **Cases** | 300 | 80 | 45 | 142 | - |
| **Age (average/range)** | 62.38 (42-83) | 63.56 (41-94) | 64.32 (44-85) | 68.44 (43-91) | 0.0997 |
| **PSA (ng/mL)** | - | - | <3 (n=8);  3-10 (n=20);  >10 (n=17) | <3 (n=11);  3-10 (n=50);  >10 (n=81) | 0.2936 |
| **fPSA/tPSA (median/range)** | - | - | 0.15 (0.06-0.39) | 0.12 (0.005-0.91) | 0.0946 |
| **BMI (kg/m^2^)** | 24.77 (2.63) | 24.69 (3.00) | 24.86 (3.45) | 23.90 (2.94) | 0.1537 |
| **HDL (mmol/L)** | 1.35 (0.29) | 1.03 (0.18) | 1.33 (0.63) | 1.21 (0.51) | 0.0801 |
| **LDL (mmol/L)** | 3.31 (0.80) | 3.01 (0.79) | 2.99 (0.74) | 2.95 (0.93) | 0.0625 |
| **TG (mmol/L)** | 1.49 (0.94) | 1.62 (0.92) | 1.49 (0.91) | 1.58 (0.96) | 0.627 |
| **TC (mmol/L)** | 5.30 (1.07) | 4.64 (0.92) | 4.84 (1.18) | 4.66 (1.03) | 0.082 |
| **Smoking status** | Yes (n=28)  No (n=272) | Yes (n=8)  No (n=72) | Yes (n=4)  No (n=41) | Yes (n=14)  No (n=128) | 0.9949 |
| **Alcohol consumption** | Yes (n=28)  No (n=272) | Yes (n=10)  No (n=70) | Yes (n=2)  No (n=43) | Yes (n=21)  No (n=121) | 0.1611 |
| **Cardiovascular disease** | Yes (n=24)  No (n=276) | Yes (n=11)  No (n=69) | Yes (n=6)  No (n=39) | Yes (n=18)  No (n=124) | 0.2604 |
| **Hypertension** | Yes (n=44)  No (n=256) | Yes (n=16)  No (n=64) | Yes (n=8)  No (n=37) | Yes (n=29)  No (n=113) | 0.4175 |
| **Diabetes** | Yes (n=22)  No (n=278) | Yes (n=12)  No (n=68) | Yes (n=6)  No (n=39) | Yes (n=20)  No (n=122) | 0.0658 |
| **Primary/Secondary** | - | - | - | Primary | - |
| **Tumor metastasis** | - | - | - | Positive (n=14);  Negative (n=128) | - |
| **TNM stage** |  |  |  | I (n=46);  II (n=62);  III (n=33);  IV (n=1) | - |
| **Gleason score** | - | - | - | 6 (n=26);  7 (n=75);  8 (n=15);  9 (n=21);  10 (n=5) | - |

***Note:*** *p value was calculated by one-way ANOVA (analysis of variance) method.*

***Table S2.*** Clinical characteristics of the subjects in the internal validation cohort.

| **Characteristics** | **Validation set** | | | | **p value** |
| --- | --- | --- | --- | --- | --- |
|  | **HC** | **UD** | **BPH** | **PCa** |  |
| **Cases** | 150 | 40 | 23 | 71 | - |
| **Age (average/range)** | 65.93 (41-87) | 67.39 (44-93) | 69.70 (49-92) | 69.61 (46-89) | 0.0926 |
| **PSA (ng/mL)** | - | - | <3 (n=1);  3-10 (n=15);  >10 (n=7) | <3 (n=7);  3-10 (n=23);  >10 (n=41) | 0.0976 |
| **fPSA/tPSA (median/range)** | - | - | 0.18 (0.03-0.36) | 0.12 (0.008-0.79) | 0.3706 |
| **BMI (kg/m^2^)** | 24.22 (2.97) | 23.68 (3.29) | 23.97 (3.19) | 24.17 (3.11) | 0.8295 |
| **HDL (mmol/L)** | 1.34 (0.30) | 1.10 (0.32) | 1.11 (0.21) | 1.24 (0.55) | 0.0788 |
| **LDL (mmol/L)** | 3.22 (0.74) | 2.92 (0.60) | 2.84 (0.84) | 2.86 (0.81) | 0.1012 |
| **TG (mmol/L)** | 1.47 (0.87) | 1.49 (0.89) | 1.53 (0.58) | 1.52 (0.83) | 0.8843 |
| **TC (mmol/L)** | 5.18 (1.04) | 4.58 (0.89) | 4.48 (1.09) | 4.58 (1.13) | 0.0810 |
| **Smoking status** | Yes (n=12)  No (n=138) | Yes (n=4)  No (n=36) | Yes (n=2)  No (n=21) | Yes (n=11)  No (n=60) | 0.3919 |
| **Alcohol consumption** | Yes (n=14)  No (n=136) | Yes (n=5)  No (n=35) | Yes (n=2)  No (n=21) | Yes (n=10)  No (n=61) | 0.7230 |
| **Cardiovascular disease** | Yes (n=18)  No (n=132) | Yes (n=4)  No (n=36) | Yes (n=3)  No (n=20) | Yes (n=8)  No (n=63) | 0.9815 |
| **Hypertension** | Yes (n=19)  No (n=131) | Yes (n=5)  No (n=35) | Yes (n=3)  No (n=20) | Yes (n=9)  No (n=62) | 0.9981 |
| **Diabetes** | Yes (n=16)  No (n=134) | Yes (n=4)  No (n=36) | Yes (n=3)  No (n=20) | Yes (n=7)  No (n=64) | 0.9776 |
| **Primary/Secondary** | - | - | - | Primary | - |
| **Tumor metastasis** | - | - | - | Positive (n=3);  Negative (n=68) | - |
| **TNM stage** |  |  |  | I (n=23);  II (n=35);  III (n=13);  IV (n=0) | - |
| **Gleason score** | - | - | - | 6 (n=10);  7 (n=39);  8 (n=9);  9 (n=8);  10 (n=5) | - |

***Note:*** *p value was calculated by one-way ANOVA (analysis of variance) method.*

***Table S3.*** Clinical characteristics of the subjects in the independent validation cohort.

| **Characteristics** | **External validation set** | | | | **p value** |
| --- | --- | --- | --- | --- | --- |
|  | **HC** | **UD** | **BPH** | **PCa** |  |
| **Cases** | 150 | 40 | 21 | 71 | - |
| **Age (average/range)** | 62.55 (47-85) | 60.15 (42-91) | 65.67 (51-83) | 69.49 (55-89) | 0.0754 |
| **PSA (ng/mL)** | - | - | <3 (n=5);  3-10 (n=9);  >10 (n=7) | <3 (n=4);  3-10 (n=31);  >10 (n=36) | 0.5172 |
| **fPSA/tPSA (median/range)** | - | - | 0.16 (0.05-0.33) | 0.12 (0.006-0.50) | 0.141 |
| **BMI (kg/m^2^)** | 24.13 (2.69) | 24.14 (2.93) | 25.32 (2.68) | 23.82 (2.69) | 0.0944 |
| **HDL (mmol/L)** | 1.34 (0.29) | 1.17 (0.24) | 1.19 (0.17) | 1.15 (0.27) | 0.0636 |
| **LDL (mmol/L)** | 3.15 (0.67) | 2.63 (0.70) | 2.89 (0.85) | 2.75 (0.73) | 0.0712 |
| **TG (mmol/L)** | 1.54 (0.91) | 1.63 (1.03) | 1.62 (0.89) | 1.51 (0.72) | 0.9198 |
| **TC (mmol/L)** | 5.06 (0.87) | 4.64 (0.92) | 4.61 (1.15) | 4.55 (1.14) | 0.0846 |
| **Smoking status** | Yes (n=14)  No (n=136) | Yes (n=3)  No (n=37) | Yes (n=3)  No (n=18) | Yes (n=5)  No (n=66) | 0.8214 |
| **Alcohol consumption** | Yes (n=18)  No (n=132) | Yes (n=5)  No (n=35) | Yes (n=2)  No (n=19) | Yes (n=9)  No (n=62) | 0.9646 |
| **Cardiovascular disease** | Yes (n=18)  No (n=132) | Yes (n=5)  No (n=35) | Yes (n=1)  No (n=20) | Yes (n=7)  No (n=64) | 0.7119 |
| **Hypertension** | Yes (n=18)  No (n=132) | Yes (n=6)  No (n=34) | Yes (n=3)  No (n=18) | Yes (n=9)  No (n=62) | 0.9679 |
| **Diabetes** | Yes (n=14)  No (n=136) | Yes (n=4)  No (n=36) | Yes (n=2)  No (n=19) | Yes (n=8)  No (n=63) | 0.9712 |
| **Primary/Secondary** | - | - | - | Primary | - |
| **Tumor metastasis** | - | - | - | Positive (n=8);  Negative (n=63) | - |
| **TNM stage** | - | - | - | I (n=25);  II (n=33);  III (n=13);  IV (n=0) | - |
| **Gleason score** | - | - | - | 6 (n=11);  7 (n=40);  8 (n=9);  9 (n=9);  10 (n=2) | - |

***Note:*** *p value was calculated by one-way ANOVA (analysis of variance) method.*

***Table S4.*** Clinical characteristics of the subjects for RT-PCR and Western Blot analysis.

| **Number** | **Age** | **Gender** | **Gleason score** | **Pathological classification** |
| --- | --- | --- | --- | --- |
| **#1** | 74 | Male | 4+3 | Prostate Adenocarcinoma |
| **#2** | 62 | Male | 4+4 | Prostate Adenocarcinoma |
| **#3** | 59 | Male | 3+4 | Prostate Adenocarcinoma |
| **#4** | 72 | Male | 4+5 | Prostate Adenocarcinoma |
| **#5** | 81 | Male | 4+4 | Prostate Adenocarcinoma |
| **#6** | 77 | Male | 3+4 | Prostate Adenocarcinoma |
| **#7** | 57 | Male | 3+4 | Prostate Adenocarcinoma |
| **#8** | 73 | Male | 4+3 | Prostate Adenocarcinoma |
| **#9** | 80 | Male | 4+3 | Prostate Adenocarcinoma |
| **#10** | 56 | Male | 4+3 | Prostate Adenocarcinoma |
| **#11** | 65 | Male | 4+3 | Prostate Adenocarcinoma |
| **#12** | 82 | Male | 4+5 | Prostate Adenocarcinoma |
| **#13** | 72 | Male | 4+5 | Prostate Adenocarcinoma |
| **#14** | 64 | Male | 3+4 | Prostate Adenocarcinoma |
| **#15** | 70 | Male | 3+4 | Prostate Adenocarcinoma |
| **#16** | 68 | Male | 3+4 | Prostate Adenocarcinoma |
| **#17** | 68 | Male | 4+3 | Prostate Adenocarcinoma |
| **#18** | 76 | Male | 4+3 | Prostate Adenocarcinoma |
| **#19** | 58 | Male | 3+4 | Prostate Adenocarcinoma |
| **#20** | 72 | Male | 4+3 | Prostate Adenocarcinoma |

***Table S5.*** Summary of feature peaks that could be used to distinguish PCa patients from healthy controls.

| **No.** | **Detected m/z** | **Identification** | **Discovery**  **p value** | **Validation**  **p value** | **Trend** |
| --- | --- | --- | --- | --- | --- |
| **1** | 102.0565 | GABA | 1.15×10^-32^ | 6.75×10^-17^ | ↓ |
| **2** | 103.0041 | Malonic acid | 5.46×10^-27^ | 8.67×10^-13^ | ↓ |
| **3** | 104.0334 | Serine | 1.07×10^-4^ | 1.01×10^-3^ | ↓ |
| **4** | 107.0561 | m-Cresol | 2.37×10^-10^ | 1.09×10^-4^ | ↓ |
| **5** | 108.0134 | Hypotaurine | 1.52×10^-3^ | 3.84×10^-2^ | ↑ |
| **6** | 109.0298 | Hydroquinone | 3.37×10^-38^ | 1.91×10^-22^ | ↓ |
| **7** | 110.0524 | Cytosine | 1.04×10^-2^ | 1.73×10^-2^ | ↑ |
| **8** | 111.0213 | Uracil | 1.57×10^-27^ | 6.87×10^-12^ | ↓ |
| **9** | 112.0495 | Creatinine | 3.66×10^-2^ | 1.62×10^-4^ | ↑ |
| **10** | 114.0574 | L-Proline | 2.06×10^-23^ | 3.78×10^-10^ | ↑ |
| **11** | 115.0047 | Maleic acid | 9.09×10^-5^ | 6.37×10^-3^ | ↑ |
| **12** | 116.0726 | L-Valine | 4.06×10^-7^ | 1.64×10^-3^ | ↑ |
| **13** | 117.0197 | Succinic acid | 4.07×10^-2^ | 3.04×10^-2^ | ↑ |
| **14** | 121.0306 | Benzoic acid | 3.24×10^-76^ | 5.80×10^-41^ | ↓ |
| **15** | 122.0255 | Nicotinic acid | 3.77×10^-8^ | 1.17×10^-8^ | ↓ |
| **16** | 124.0079 | Taurine | 9.23×10^-8^ | 5.45×10^-9^ | ↑ |
| **17** | 125.0222 | 5-Methylfuran-2-carboxylic acid | 1.08×10^-17^ | 2.84×10^-15^ | ↑ |
| **18** | 129.0201 | Citraconic acid | 2.35×10^-2^ | 9.57×10^-5^ | ↑ |
| **19** | 130.0517 | N-Acetylalanine | 5.57×10^-5^ | 4.15×10^-4^ | ↑ |
| **20** | 132.0312 | L-Aspartic acid | 6.70×10^-10^ | 3.49×10^-5^ | ↑ |
| **21** | 137.0240 | Salicylic acid | 1.38×10^-56^ | 1.55×10^-32^ | ↓ |
| **22** | 139.0529 | Imidazolepropionic acid | 2.03×10^-4^ | 3.75×10^-3^ | ↑ |
| **23** | 142.0886 | Proline betaine | 1.11×10^-3^ | 2.85×10^-2^ | ↓ |
| **24** | 143.1098 | Caprylic acid | 1.96×10^-3^ | 2.32×10^-2^ | ↑ |
| **25** | 144.0651 | Allysine | 8.58×10^-58^ | 2.22×10^-34^ | ↓ |
| **26** | 145.0627 | Glutamine | 2.18×10^-6^ | 7.65×10^-7^ | ↑ |
| **27** | 146.0445 | Glutamic acid | 2.84×10^-5^ | 2.46×10^-3^ | ↓ |
| **28** | 148.0455 | L-Methionine | 1.87×10^-10^ | 4.46×10^-5^ | ↓ |
| **29** | 149.0247 | Phenylglyoxylic acid | 1.72×10^-15^ | 2.26×10^-7^ | ↓ |
| **30** | 151.0256 | Xanthine | 6.85×10^-11^ | 8.06×10^-10^ | ↓ |
| **31** | 152.0366 | 3-Hydroxyanthranilic acid | 8.05×10^-9^ | 7.10×10^-6^ | ↑ |
| **32** | 153.0220 | Gentisic acid | 2.19×10^-17^ | 1.07×10^-9^ | ↓ |
| **33** | 154.0604 | L-Histidine | 5.33×10^-11^ | 4.45×10^-7^ | ↑ |
| **34** | 155.0467 | Imidazolelactic acid | 1.62×10^-5^ | 6.44×10^-3^ | ↑ |
| **35** | 156.0660 | N-Acetylproline | 3.94×10^-34^ | 8.85×10^-13^ | ↑ |
| **36** | 157.0255 | L-Dihydroorotic acid | 1.20×10^-2^ | 3.46×10^-2^ | ↑ |
| **37** | 158.0835 | N-Acetylvaline | 2.61×10^-23^ | 8.67×10^-14^ | ↓ |
| **38** | 164.0723 | Phenylalanine | 2.38×10^-4^ | 8.79×10^-3^ | ↓ |
| **39** | 165.0191 | Phthalic acid | 7.31×10^-15^ | 3.68×10^-7^ | ↓ |
| **40** | 167.0221 | Uric acid | 5.03×10^-14^ | 2.75×10^-7^ | ↑ |
| **41** | 167.9981 | Cysteic acid | 1.03×10^-7^ | 1.28×10^-4^ | ↑ |
| **42** | 170.0442 | N-Acetylglutamic acid | 3.85×10^-2^ | 1.96×10^-2^ | ↑ |
| **43** | 173.1033 | L-Arginine | 1.41×10^-6^ | 1.33×10^-4^ | ↑ |
| **44** | 174.0893 | Citrulline | 5.86×10^-6^ | 5.59×10^-5^ | ↑ |
| **45** | 178.0621 | Hippuric acid | 4.67×10^-5^ | 2.76×10^-3^ | ↑ |
| **46** | 190.0550 | N-Acetylmethionine | 4.05×10^-9^ | 1.26×10^-5^ | ↑ |
| **47** | 207.1728 | 5-Tetradecenoic acid | 6.35×10^-11^ | 2.36×10^-4^ | ↑ |
| **48** | 283.2565 | Stearic acid | 8.87×10^-20^ | 4.30×10^-12^ | ↑ |

***Note:*** *p value was calculated by student’s t test and corrected by Benjamini-Hochberg method.*

***Table S6.*** Summary of feature peaks that could be used to distinguish BPH patients from healthy controls.

| **No.** | **Detected m/z** | **Identification** | **Discovery**  **p value** | **Validation**  **p value** | **Trend** |
| --- | --- | --- | --- | --- | --- |
| **1** | 102.0565 | GABA | 7.20×10^-26^ | 8.96×10^-14^ | ↓ |
| **2** | 103.0041 | Malonic acid | 8.10×10^-26^ | 3.13×10^-26^ | ↓ |
| **3** | 104.0334 | Serine | 1.35×10^-4^ | 1.26×10^-3^ | ↓ |
| **4** | 107.0561 | m-Cresol | 5.51×10^-6^ | 3.15×10^-6^ | ↓ |
| **5** | 109.0298 | Hydroquinone | 1.95×10^-16^ | 7.59×10^-10^ | ↓ |
| **6** | 111.0213 | Uracil | 3.00×10^-12^ | 3.31×10^-8^ | ↓ |
| **7** | 114.0574 | L-Proline | 8.13×10^-10^ | 2.30×10^-2^ | ↑ |
| **8** | 116.0726 | L-Valine | 1.75×10^-10^ | 5.48×10^-6^ | ↑ |
| **9** | 117.0197 | Succinic acid | 2.80×10^-4^ | 8.08×10^-5^ | ↑ |
| **10** | 118.0516 | L-Threonine | 2.24×10^-4^ | 1.08×10^-3^ | ↑ |
| **11** | 121.0306 | Benzoic acid | 2.31×10^-52^ | 6.18×10^-25^ | ↓ |
| **12** | 122.0255 | Nicotinic acid | 1.07×10^-13^ | 1.58×10^-3^ | ↓ |
| **13** | 124.0079 | Taurine | 3.45×10^-4^ | 6.72×10^-6^ | ↑ |
| **14** | 125.0222 | 5-Methylfuran-2-carboxylic acid | 1.66×10^-6^ | 6.52×10^-7^ | ↑ |
| **15** | 128.0355 | L-Pyroglutamic acid | 7.52×10^-5^ | 1.10×10^-3^ | ↓ |
| **16** | 132.0312 | L-Aspartic acid | 2.76×10^-9^ | 1.62×10^-8^ | ↑ |
| **17** | 137.0240 | Salicylic acid | 1.46×10^-33^ | 3.41×10^-19^ | ↓ |
| **18** | 139.0529 | Imidazolepropionic acid | 5.64×10^-18^ | 9.54×10^-10^ | ↑ |
| **19** | 142.0886 | Proline betaine | 9.94×10^-3^ | 2.73×10^-3^ | ↓ |
| **20** | 143.1098 | Caprylic acid | 5.39×10^-15^ | 3.24×10^-7^ | ↑ |
| **21** | 144.0651 | Allysine | 3.02×10^-32^ | 4.89×10^-21^ | ↓ |
| **22** | 145.0627 | Glutamine | 3.21×10^-2^ | 1.11×10^-4^ | ↑ |
| **23** | 146.0445 | Glutamic acid | 1.92×10^-5^ | 2.01×10^-3^ | ↓ |
| **24** | 148.0455 | L-Methionine | 2.38×10^-14^ | 7.25×10^-4^ | ↓ |
| **25** | 149.0247 | Phenylglyoxylic acid | 3.87×10^-8^ | 2.20×10^-4^ | ↓ |
| **26** | 151.0256 | Xanthine | 1.01×10^-8^ | 2.56×10^-5^ | ↓ |
| **27** | 153.0220 | Gentisic acid | 6.58×10^-11^ | 1.66×10^-5^ | ↓ |
| **28** | 154.0604 | L-Histidine | 2.03×10^-4^ | 7.76×10^-5^ | ↑ |
| **29** | 155.0467 | Imidazolelactic acid | 1.00×10^-19^ | 1.29×10^-10^ | ↑ |
| **30** | 156.0660 | N-Acetylproline | 2.33×10^-34^ | 1.28×10^-14^ | ↑ |
| **31** | 157.0255 | L-Dihydroorotic acid | 2.17×10^-7^ | 6.19×10^-7^ | ↑ |
| **32** | 158.0835 | N-Acetylvaline | 1.94×10^-13^ | 4.06×10^-8^ | ↓ |
| **33** | 160.0620 | N-Acetylthreonine | 5.41×10^-5^ | 1.39×10^-4^ | ↑ |
| **34** | 164.0723 | Phenylalanine | 9.52×10^-7^ | 6.37×10^-3^ | ↓ |
| **35** | 165.0191 | Phthalic acid | 4.55×10^-8^ | 2.79×10^-4^ | ↓ |
| **36** | 167.0221 | Uric acid | 1.15×10^-12^ | 4.43×10^-9^ | ↑ |
| **37** | 167.9981 | Cysteic acid | 4.07×10^-7^ | 7.41×10^-5^ | ↑ |
| **38** | 173.1033 | L-Arginine | 1.47×10^-4^ | 9.89×10^-4^ | ↑ |
| **39** | 178.0621 | Hippuric acid | 2.11×10^-8^ | 2.70×10^-11^ | ↑ |
| **40** | 180.0681 | L-Tyrosine | 8.39×10^-9^ | 3.10×10^-6^ | ↑ |
| **41** | 190.0550 | N-Acetylmethionine | 1.24×10^-4^ | 3.92×10^-4^ | ↑ |
| **42** | 203.0829 | L-Tryptophan | 1.15×10^-2^ | 1.27×10^-3^ | ↑ |
| **43** | 207.1728 | 5-Tetradecenoic acid | 2.95×10^-15^ | 1.56×10^-8^ | ↑ |
| **44** | 283.2565 | Stearic acid | 5.33×10^-34^ | 2.81×10^-13^ | ↑ |

***Note:*** *p value was calculated by one-way ANOVA (analysis of variance) method.*

***Table S7.*** Summary of feature peaks that could be used to distinguish PCa patients from BPH subjects.

| **No.** | **Detected m/z** | **Identification** | **Discovery**  **p value** | **Validation**  **p value** | **Trend** |
| --- | --- | --- | --- | --- | --- |
| **1** | 102.0565 | GABA | 5.71×10^-3^ | 9.22×10^-3^ | ↑ |
| **2** | 103.0041 | Malonic acid | 3.82×10^-2^ | 7.54×10^-3^ | ↑ |
| **3** | 110.0524 | Cytosine | 4.20×10^-2^ | 6.48×10^-3^ | ↑ |
| **4** | 112.0495 | Creatinine | 3.60×10^-2^ | 7.09×10^-3^ | ↑ |
| **5** | 114.0574 | L-Proline | 4.31×10^-2^ | 4.50×10^-4^ | ↑ |
| **6** | 128.0355 | L-Pyroglutamic acid | 4.17×10^-2^ | 2.18×10^-3^ | ↑ |
| **7** | 139.0529 | Imidazolepropionic acid | 2.78×10^-3^ | 4.12×10^-5^ | ↓ |
| **8** | 143.1098 | Caprylic acid | 3.22×10^-3^ | 6.02×10^-3^ | ↓ |
| **9** | 144.0651 | Allysine | 3.93×10^-2^ | 1.46×10^-3^ | ↑ |
| **10** | 146.0445 | Glutamic acid | 3.06×10^-2^ | 3.22×10^-2^ | ↑ |
| **11** | 148.0455 | L-Methionine | 1.84×10^-4^ | 3.07×10^-2^ | ↑ |
| **12** | 155.0467 | Imidazolelactic acid | 1.86×10^-3^ | 2.22×10^-4^ | ↓ |
| **13** | 156.0660 | N-Acetylproline | 4.15×10^-2^ | 1.78×10^-2^ | ↓ |
| **14** | 157.0255 | L-Dihydroorotic acid | 4.73×10^-2^ | 9.58×10^-3^ | ↓ |
| **15** | 158.0835 | N-Acetylvaline | 3.71×10^-2^ | 2.93×10^-2^ | ↑ |
| **16** | 159.0681 | Pimelic acid | 3.78×10^-2^ | 1.59×10^-2^ | ↓ |
| **17** | 160.0620 | N-Acetylthreonine | 4.63×10^-2^ | 8.98×10^-3^ | ↓ |
| **18** | 165.0191 | Phthalic acid | 1.82×10^-2^ | 1.34×10^-2^ | ↑ |
| **19** | 180.0681 | L-Tyrosine | 3.95×10^-2^ | 1.98×10^-3^ | ↓ |
| **20** | 190.0550 | N-Acetylmethionine | 3.26×10^-2^ | 1.20×10^-2^ | ↑ |

***Table S8.*** LC-MS/MS identification of potential feature peaks for discrimination of patients with prostate disease.

| **No.** | **Measured *m/z*** | **Theoretical *m/z*** | **Adductive ions** | **Identification** | **Delta** **(ppm)** | **Main fragments** | **Database** |
| --- | --- | --- | --- | --- | --- | --- | --- |
| **1** | 102.0581 | 102.0561 | M-H | GABA | 20 | 58,84 | HMDB |
| **2** | 103.0042 | 103.0037 | M-H | Malonic acid | 5 | 41,59,85 | HMDB |
| **3** | 104.0368 | 104.0353 | M-H | Serine | 14 | 43,56,74 | HMDB |
| **4** | 107.0524 | 107.0502 | M-H | m-Cresol | 20 | 65,79,91 | HMDB |
| **5** | 110.0260 | 110.0270 | M+H | Hypotaurine | 9 | 69,81,92 | HMDB |
| **6** | 109.0308 | 109.0295 | M-H | Hydroquinone | 12 | 41,53,55,65,81 | HMDB |
| **7** | 112.0519 | 112.0505 | M+H | Cytosine | 12 | 52,67,68,69,95 | HMDB |
| **8** | 111.0217 | 111.0200 | M-H | Uracil | 15 | 42,50,68,94 | HMDB |
| **9** | 114.0668 | 114.0662 | M+H | Creatinine | 5 | 44,55,72,86 | HMDB |
| **10** | 116.0715 | 116.0706 | M+H | L-Proline | 8 | 54,55,56,70,71,98 | HMDB |
| **11** | 115.0043 | 115.0037 | M-H | Maleic acid | 5 | 45,53,69,71,97 | HMDB |
| **12** | 118.0866 | 118.0863 | M+H | L-Valine | 3 | 56,57,60,72,74 | HMDB |
| **13** | 117.0204 | 117.0193 | M-H | Succinic acid | 9 | 45,55,71,73,81,99 | HMDB |
| **14** | 120.0661 | 120.0655 | M+H | L-Threonine | 5 | 56,58,74,102 | HMDB |
| **15** | 121.0299 | 121.0295 | M-H | Benzoic acid | 3 | 49,51,77,103 | HMDB |
| **16** | 122.0255 | 122.0248 | M-H | Nicotinic acid | 6 | 78,95,96 | HMDB |
| **17** | 126.0224 | 126.0219 | M+H | Taurine | 4 | 84,98,109,115 | HMDB |
| **18** | 125.0259 | 125.0244 | M-H | 5-Methylfuran-2-carboxylic acid | 12 | 63,65,81,89,107 | HMDB |
| **19** | 128.0365 | 128.0353 | M-H | L-Pyroglutamic acid | 9 | 52,59,72,82 | HMDB |
| **20** | 129.0218 | 129.0193 | M-H | Citraconic acid | 19 | 65,85,111 | HMDB |
| **21** | 130.0527 | 130.0510 | M-H | N-Acetylalanine | 13 | 58,70,82,86,88,114 | HMDB |
| **22** | 134.0458 | 134.0448 | M+H | L-Aspartic acid | 8 | 77,79,89,90,116 | HMDB |
| **23** | 137.0240 | 137.0244 | M-H | Salicylic acid | 3 | 65,67,75,93 | HMDB |
| **24** | 139.0523 | 139.0513 | M-H | Imidazolepropionic acid | 7 | 41,67,95,112,121 | HMDB |
| **25** | 142.0897 | 142.0874 | M-H | Proline betaine | 17 | 45,53,59,69,83 | HMDB |
| **26** | 143.1088 | 143.1078 | M-H | Caprylic acid | 7 | 45,53,57,67,81,95,107,125 | HMDB |
| **27** | 144.0677 | 144.0666 | M-H | Allysine | 8 | 45,53,74,82,100,102,126 | HMDB |
| **28** | 145.0631 | 145.0619 | M-H | Glutamine | 9 | 42,58,74,82,109,127 | HMDB |
| **29** | 148.0617 | 148.0604 | M+H | Glutamic acid | 9 | 59,75,102,115 | HMDB |
| **30** | 148.0436 | 148.0438 | M-H | L-Methionine | 1 | 47,73,86,100,115 | HMDB |
| **31** | 149.0290 | 149.0244 | M-H | Phenylglyoxylic acid | 31 | 65,77,89,103,105 | HMDB |
| **32** | 151.0278 | 151.0261 | M-H | Xanthine | 11 | 42,53,81,108,124,133 | HMDB |
| **33** | 154.0510 | 154.0499 | M+H | 3-Hydroxyanthranilic acid | 7 | 53,54,80,92,108,136 | HMDB |
| **34** | 153.0206 | 153.0193 | M-H | Gentisic acid | 8 | 53,69,81,108 | HMDB |
| **35** | 154.0627 | 154.0622 | M-H | L-Histidine | 3 | 67,80,81,93,110,137 | HMDB |
| **36** | 155.0470 | 155.0462 | M-H | Imidazolelactic acid | 5 | 40,67,81,111 | HMDB |
| **37** | 156.0663 | 156.0666 | M-H | N-Acetylproline | 2 | 45,83,96,114 | HMDB |
| **38** | 157.0263 | 157.0255 | M-H | L-Dihydroorotic acid | 5 | 42,72,113,129,139 | HMDB |
| **39** | 158.0821 | 158.0823 | M-H | N-Acetylvaline | 1 | 41,58,98,116 | HMDB |
| **40** | 159.0666 | 159.0663 | M-H | Pimelic acid | 2 | 59,69,97,115,141 | HMDB |
| **41** | 160.0621 | 160.0615 | M-H | N-Acetylthreonine | 4 | 58,74,98,116,118,142 | HMDB |
| **42** | 164.0729 | 164.0717 | M-H | Phenylalanine | 7 | 72,77,92,103,120,147 | HMDB |
| **43** | 165.0215 | 165.0193 | M-H | Phthalic acid | 13 | 51,77,93,95,103,121,147 | HMDB |
| **44** | 167.0223 | 167.0211 | M-H | Uric acid | 7 | 42,69,81,96,124,149 | HMDB |
| **45** | 167.9990 | 167.9972 | M-H | Cysteic acid | 11 | 72,81,88,95,107,124,150 | HMDB |
| **46** | 170.0461 | 170.0453 | M-H_2_O-H | N-Acetylglutamic acid | 5 | 58,72,85,100,128,144,146 | HMDB |
| **47** | 173.1060 | 173.1044 | M-H | L-Arginine | 9 | 41,69,72,81,112,131,155 | HMDB |
| **48** | 174.0882 | 174.0884 | M-H | Citrulline | 1 | 42,59,66,83,85,114,131 | HMDB |
| **49** | 178.0512 | 178.0510 | M-H | Hippuric acid | 1 | 77,100,105,107,132,134 | HMDB |
| **50** | 180.0674 | 180.0666 | M-H | L-Tyrosine | 4 | 72,93,119 | HMDB |
| **51** | 190.0562 | 190.0543 | M-H | N-Acetylmethionine | 10 | 58,116,128,130,148,172 | HMDB |
| **52** | 203.0837 | 203.0826 | M-H | L-Tryptophan | 5 | 72,74,116,130,142 | HMDB |
| **53** | 207.1761 | 207.1749 | M-H_2_O-H | 5-Tetradecenoic acid | 6 | 45,59,71,97,109,125,135,139,149,163 | HMDB |
| **54** | 283.2661 | 283.2643 | M-H | Stearic acid | 7 | 99,125,139,155,183,197,211,239,265 | HMDB |

***Table S9.*** MALDI-TOF/TOF tandem mass spectrometry identification of urine metabolites.

| **No.** | **Identification** | **Detected m/z in urine** | **MS/MS fragments of urine metabolites in MALDI** | **Detected m/z of standard** | **MS/MS fragments of standard in MALDI** | **Possible metabolites** | **Molecular formula** | **Theoretical m/z** |
| --- | --- | --- | --- | --- | --- | --- | --- | --- |
| **1** | standard* | 102.0565 | 42,58,71,84 | 102.0560 | 58,71,84 | GABA | C_4_H_9_NO_2_ | 102.0561 |
| **2** | standard* | 103.0041 | 41,44,59,72,75,85 | 103.0040 | 41,44,59,72,75,85 | Malonic acid | C_3_H_4_O_4_ | 103.0037 |
| **3** | standard* | 104.0334 | 58,60,74 | 104.0349 | 58,60,74 | Serine | C_3_H_7_NO_3_ | 104.0353 |
| **4** | standard | 107.0561 | 41,65,79,81,84 | 107.0497 | 41,65,79,81,84 | m-Cresol | C_7_H_8_O | 107.0502 |
| **5** | standard* | 108.0134 | 64,65,90 | 108.0127 | 64,65,90 | Hypotaurine | C_2_H_7_NO_2_S | 108.0125 |
| **6** | standard* | 109.0298 | 41, 65,81,83 | 109.0294 | 41,65,81,83 | Hydroquinone | C_6_H_6_O_2_ | 109.0295 |
| **7** | standard* | 110.0524 | 42,67,84 | 110.0357 | 42,67,84 | Cytosine | C_4_H_5_N_3_O | 110.0360 |
| **8** | standard* | 111.0213 | 42,68,84,85 | 111.0202 | 42,68,84,85,94 | Uracil | C_4_H_4_N_2_O_2_ | 111.0200 |
| **9** | standard* | 112.0495 | 41,68,85,95 | 112.0510 | 41,68,85,95 | Creatinine | C4H7N3O | 112.0516 |
| **10** | standard* | 114.0574 | 45,59,68,86 | 114.0561 | 45,59,68,86 | L-Proline | C_5_H_9_NO_2_ | 114.0561 |
| **11** | Standard* | 115.0047 | 45,53,69 | 115.0044 | 45,53,69,71 | Maleic acid | C_4_H_4_O_4_ | 115.0037 |
| **12** | standard* | 116.0726 | 45,70,75,89,93,99 | 116.0721 | 45,70,75,89,93 | L-Valine | C_5_H_11_NO_2_ | 116.0717 |
| **13** | standard* | 117.0197 | 73,90,99 | 117.0190 | 61,73,90,99 | Succinic acid | C_4_H_6_O_4_ | 117.0193 |
| **14** | standard* | 118.0516 | 56,72,74 | 118.0507 | 56,72,74 | L-Threonine | C_4_H_9_NO_3_ | 118.0510 |
| **15** | standard* | 121.0306 | 49,77,103 | 121.0297 | 49,77,103 | Benzoic acid | C_7_H_6_O_2_ | 121.0295 |
| **16** | standard | 122.0255 | 45,77,78,95,100,105 | 122.0243 | 77,78,95,100,105 | Nicotinic acid | C_6_H_5_NO_2_ | 122.0248 |
| **17** | standard* | 124.0079 | 60,65,79,80,95,105,107 | 124.0081 | 60,65,79,80,95,105 | Taurine | C_2_H_7_NO_3_S | 124.0074 |
| **18** | standard* | 125.0222 | 41,81,107 | 125.0239 | 41,81,107 | 5-Methylfuran-2-carboxylic acid | C_6_H_6_O_3_ | 125.0244 |
| **19** | standard* | 128.0355 | 82,84,102,103,110 | 128.0356 | 71,82,84,102,103,110 | L-Pyroglutamic acid | C_5_H_7_NO_3_ | 128.0353 |
| **20** | standard* | 129.0201 | 27,36,43,59,88,113 | 129.0194 | 27,36,43,59,88,113 | Citraconic acid | C_5_H_6_O_4_ | 129.0193 |
| **21** | standard* | 130.0517 | 42,70,88,112 | 130.0511 | 42,70,88,112 | N-Acetylalanine | C_5_H_9_NO_3_ | 130.0510 |
| **22** | standard* | 132.0312 | 42,71,72,88,115 | 132.0304 | 42,71,72,88 | L-Aspartic acid | C_4_H_7_NO_4_ | 132.0302 |
| **23** | standard* | 137.0240 | 79,93,119 | 137.0248 | 41,79,93,119 | Salicylic acid | C_7_H_6_O_3_ | 137.0244 |
| **24** | standard* | 139.0529 | 67,95,121 | 139.0520 | 41,67,95,112,121 | Imidazolepropionic acid | C_6_H_8_N_2_O_2_ | 139.0513 |
| **25** | standard* | 142.0886 | 45,53,96,99,115,121 | 142.0870 | 45,53,83,85,96,99,115,121 | Proline betaine | C_7_H_13_NO_2_ | 142.0874 |
| **26** | standard* | 143.1098 | 57,99,107,115,125 | 143.1076 | 57,99,107,115,125 | Caprylic acid | C_8_H_16_O_2_ | 143.1078 |
| **27** | standard* | 144.0651 | 41,100,116,126 | 144.0622 | 41,100,108,116,126 | Allysine | C_6_H_11_NO_3_ | 144.0666 |
| **28** | standard* | 145.0627 | 73,101,109,127 | 145.0622 | 73,83,101,109,127 | Glutamine | C_5_H_10_N_2_O_3_ | 145.0619 |
| **29** | standard* | 146.0445 | 42,74,102,128 | 146.0461 | 74,102,128 | Glutamic acid | C_5_H_9_NO_4_ | 146.0459 |
| **30** | standard* | 148.0455 | 47,100,123 | 148.0441 | 47,100,123 | Methionine | C_5_H_11_NO_2_S | 148.0438 |
| **31** | standard* | 149.0247 | 77,89,103,105 | 149.0242 | 77,89,105,121 | Phenylglyoxylic acid | C_8_H_6_O_3_ | 149.0244 |
| **32** | standard* | 151.0256 | 42,81,108,133 | 151.0244 | 108,124,133 | Xanthine | C_5_H_4_N_4_O_2_ | 151.0261 |
| **33** | standard* | 152.0366 | 78,92,108,126,134 | 152.0348 | 78,108,126,134 | 3-Hydroxyanthranilic acid | C_7_H_7_NO_3_ | 152.0353 |
| **34** | standard* | 153.0220 | 41,81,108,123,137 | 153.0196 | 41,108,123,137 | Gentisic acid | C_7_H_6_O_4_ | 153.0193 |
| **35** | standard* | 154.0604 | 81,93,110,137 | 154.0623 | 81,93,110,137 | L-Histidine | C_6_H_9_N_3_O_2_ | 154.0622 |
| **36** | standard* | 155.0467 | 40,67,81 | 155.0461 | 40,67,81 | Imidazolelactic acid | C_6_H_8_N_2_O_3_ | 155.0462 |
| **37** | standard* | 156.0660 | 43,58,96,114,139 | 156.0667 | 43,96,114,139 | N-Acetylproline | C_7_H_11_NO_3_ | 156.0666 |
| **38** | standard* | 157.0255 | 42,113,129,130,139 | 157.0255 | 42,113,129,139 | L-Dihydroorotic acid | C_5_H_6_N_2_O_4_ | 157.0255 |
| **39** | standard* | 158.0835 | 34,41,78,99,114,116 | 158.0822 | 34,41,78,99,114,116 | N-Acetylvaline | C_7_H_13_NO_3_ | 158.0823 |
| **40** | standard* | 159.0681 | 97,115,137,141 | 159.0667 | 61,97,115,137,141 | Pimelic acid | C_7_H_12_O_4_ | 159.0663 |
| **41** | standard* | 160.0620 | 100,116,118,142 | 160.0617 | 100,116,118,142 | N-Acetylthreonine | C_6_H_11_NO_4_ | 160.0615 |
| **42** | standard* | 164.0723 | 91,103,120,147 | 164.0720 | 72,91,103,120,147 | Phenylalanine | C_9_H_11_NO_2_ | 164.0717 |
| **43** | standard* | 165.0191 | 51,77,121,147 | 165.0193 | 77,103,121,147 | Phthalic acid | C_8_H_6_O_4_ | 165.0193 |
| **44** | standard* | 167.0221 | 97,110,124,128,140,151 | 167.0214 | 69,97,124,128,140 | Uric acid | C_5_H_4_N_4_O_3_ | 167.0211 |
| **45** | standard* | 167.9981 | 81,86,124,150,151 | 167.9979 | 86,124,150,151 | Cysteic acid | C_3_H_7_NO_5_S | 167.9972 |
| **46** | standard* | 170.0442 | 100,128,144 | 170.0414 | 58,100,128,144 | N-Acetylglutamic acid | C_7_H_11_NO_5_ | 170.0453 |
| **47** | standard | 173.1033 | 77,114,131,156 | 173.1047 | 77,114,131,156 | L-Arginine | C_6_H_14_N_4_O_2_ | 173.1044 |
| **48** | standard | 174.0893 | 118,131,145,147 | 174.0879 | 118,131,145,147,157 | Citrulline | C_6_H_13_N_3_O_3_ | 174.0884 |
| **49** | standard* | 178.0621 | 77,107,132,134 | 178.0515 | 77,107,132,134 | Hippuric acid | C_9_H_9_NO_3_ | 178.0510 |
| **50** | standard* | 180.0681 | 72,93,119,139,163 | 180.0676 | 72,93,119,139 | L-Tyrosine | C_9_H_11_NO_3_ | 180.0666 |
| **51** | standard | 190.0550 | 72,129,142,148 | 190.0542 | 72,115,129,142,148 | N-Acetylmethionine | C_7_H_13_NO_3_S | 190.0543 |
| **52** | standard* | 203.0829 | 74,116,142,159,174 | 203.0822 | 74,142,159,174 | L-Tryptophan | C_11_H_12_N_2_O_2_ | 203.0826 |
| **53** | standard* | 207.1728 | 111,125,135,139,149,163 | 207.1722 | 111,125,135,149,163 | 5-Tetradecenoic acid | C_14_H_26_O_2_ | 207.1749 |
| **54** | standard* | 283.2565 | 155,183,197,211,223,239,265 | 283.2768 | 155,183,197,211,223,239,265 | Stearic acid | C_18_H_36_O_2_ | 283.2643 |

Note: Because the ion source of two MS technologies (UPLC-MS and MALDI-TOF MS) are different, so the tandem MS results are not comparable, * represents the MALDI -TOF/TOF tandem mass spectrometry results of some metabolites are consistent with the UPLC-MS/MS data.
